# Thy-1 dependent uptake of mesenchymal stem cell-derived extracellular vesicles blocks myofibroblastic differentiation

**DOI:** 10.1038/s41598-017-18288-9

**Published:** 2017-12-22

**Authors:** Tzu-Pin Shentu, Tse-Shun Huang, Mateja Cernelc-Kohan, Joy Chan, Simon S. Wong, Celia R. Espinoza, Chunting Tan, Irene Gramaglia, Henri van der Heyde, Shu Chien, James S. Hagood

**Affiliations:** 1 Department of Pediatrics, Division of Respiratory Medicine, University of California-San Diego, La Jolla, California, USA; 2 Institute of Engineering in Medicine, University of California at San Diego, La Jolla, California, USA; 3 UCLA Medical Center, Los Angeles, California, USA; 4 La Jolla Infectious Disease Institute, San Diego, California, USA; 5 Rady Children’s Hospital of San Diego, San Diego, California, USA

## Abstract

Bone marrow-derived mesenchymal stem cells (MSC) have been promoted for multiple therapeutic applications. Many beneficial effects of MSCs are paracrine, dependent on extracellular vesicles (EVs). Although MSC-derived EVs (mEVs) are beneficial for acute lung injury and pulmonary fibrosis, mechanisms of mEV uptake by lung fibroblasts and their effects on myofibroblastic differentiation have not been established. We demonstrate that mEVs, but not fibroblast EVs (fEVs), suppress TGFβ1-induced myofibroblastic differentiation of normal and idiopathic pulmonary fibrosis (IPF) lung fibroblasts. MEVs display increased time- and dose-dependent cellular uptake compared to fEVs. Removal or blocking of Thy-1, or blocking Thy-1-beta integrin interactions, decreased mEV uptake and prevented suppression of myofibroblastic differentiation. MicroRNAs (miRs) 199a/b-3p, 21-5p, 630, 22-3p, 196a-5p, 199b-5p, 34a-5p and 148a-3p are selectively packaged in mEVs. *In silico* analyses indicated that IPF lung fibroblasts have increased expression of genes that are targets of mEV-enriched miRs. MiR-630 mimics blocked TGFβ1 induction of *CDH2* in normal and IPF fibroblasts, and antagomiR-630 abrogated the effect of mEV on *CDH2* expression. These data suggest that the interaction of Thy-1 with beta integrins mediates mEV uptake by lung fibroblasts, which blocks myofibroblastic differentiation, and that mEVs are enriched for miRs that target profibrotic genes up-regulated in IPF fibroblasts.

## Introduction

Human mesenchymal stem cell-derived extracellular vesicles (mEVs) have emerged as a new therapeutic strategy for many diseases^1–3^. The beneficial effects are similar to those of their parental cells^4,5^. Extracellular vesicles (EVs) are membrane-bound vesicles secreted from cells. Current terminology refers to smaller EVs (40-100 nm), which originate from multivesicular endosomal bodies as exosomes and larger ones (100–1000 nm), which bud from the plasma membrane as microvesicles^1–3^. However, current isolation technology cannot consistently separate these subsets. Because of this technical limitation, we use the term “extracellular vesicles” as suggested by the International Society of Extracellular Vesicles^6,7^. EVs are comprised of mRNAs, non-coding RNAs, proteins and membrane lipids derived from donor cells. EVs can regulate cell proliferation, tissue repair, and regeneration^8,9^. *In vivo* therapeutic effects of mEVs have been shown in acute lung injury^10,11^, acute and chronic kidney injury^12–15^, myocardial ischemia/infarction^16–18^, pulmonary hypertension^19^ and silica-induced pulmonary fibrosis^20^. Horizontal microRNA (miR) transfer^21,22^ appears to be important in mEV-mediated tissue recovery^10,23^. It is less well known, however, the degree to which the cellular responses are dependent on mEV uptake by recipient cells.

Several routes of EV uptake have been shown in different cell types^24^. Initial protein interactions through tetraspanins, integrins and immunoglobulins, proteoglycans or lectins facilitate subsequent endocytosis into cells. Inhibition of endocytosis pathways, either through lipid raft-dependent mechanisms^25^, clathrin or macropinocytosis^26^ represses EV uptake. Here, we test the hypothesis that cellular uptake of mEV by fibroblasts is important for modulating the profibrotic myofibroblast phenotype. The cell surface protein Thy-1 (CD90) is highly expressed on the MSC cell surface, and we previously showed that Thy-1 could be shed from cell membranes in the form of vesicles^27^. Thy-1 resides in lipid rafts and has both integrin-binding (RLD) and syndecan-4 binding motifs (REKRK), consistent with its role in cell-matrix or cell-cell interactions^28^. Thy-1-integrin interaction signals heterotypically or homotypically, contributing to rigidity sensing by fibroblasts^29^ and inhibition of contraction-induced latent TGFβ1 activation and myofibroblastic differentiation^30^. Hence, we explored the role of Thy-1 in mEV binding and uptake by fibroblasts. Additionally, we identified miRs sorted into mEVs and the relevant molecular targets modulated by mEV miRs in fibrotic fibroblasts.

## Results

### MSC-derived, but not fibroblast-derived EVs modulate TGFβ1-induced myofibroblastic differentiation in a Thy-1 dependent manner

**Figure 1:**
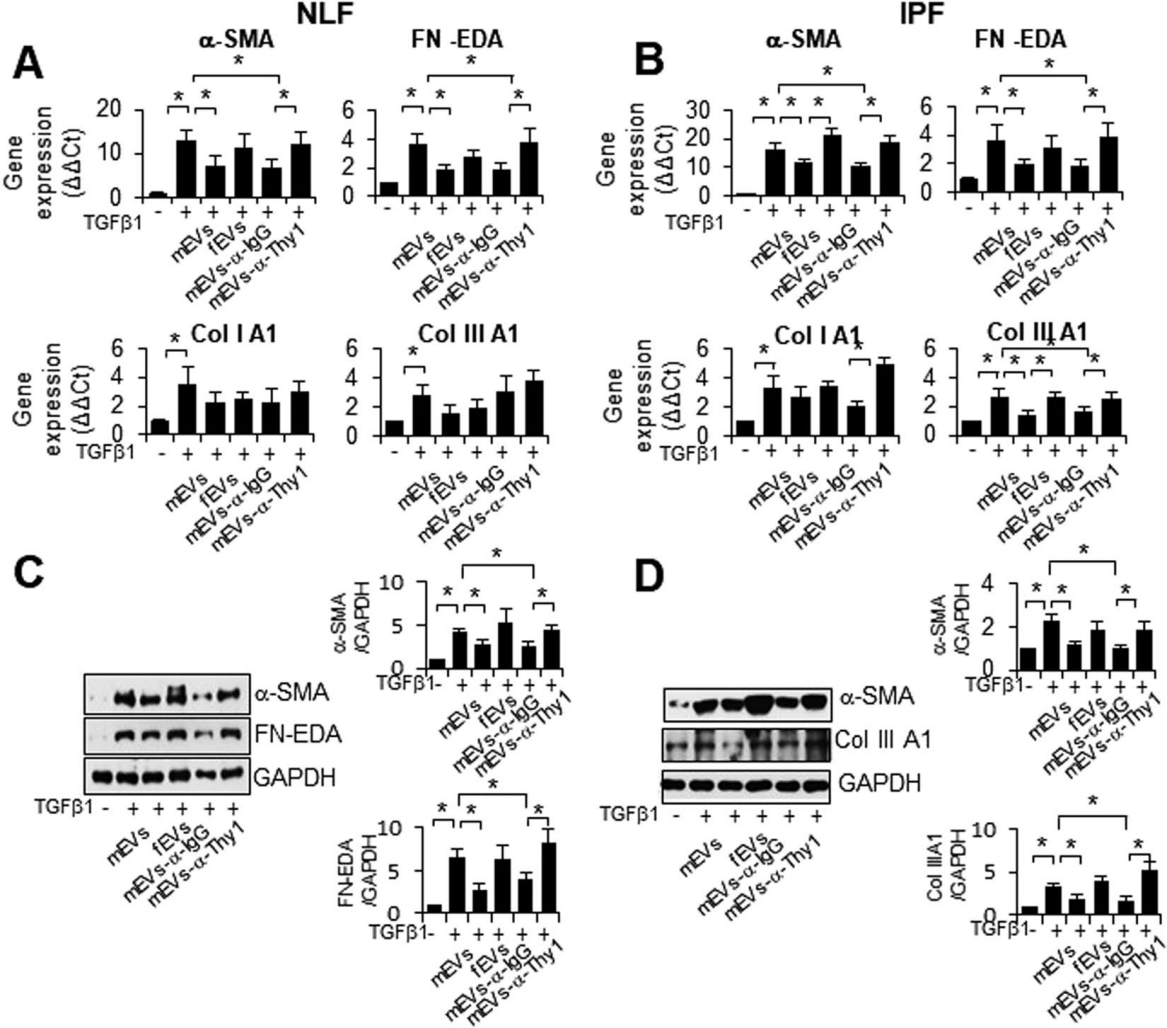
mEVs modulate TGFβ1-induced myofibroblastic differentiation in a Thy-1-dependent manner. (**A**) Normal lung fibroblasts (NLF) or (**B**) IPF lung fibroblasts were made quiescent in serum free medium for 16 hrs and then incubated with TGF-β1 (2ng/ml, overnight), together with indicated EV preparations, and total RNA was subjected to RT-PCR using primers for human α-SMA, FN-EDA, collagen I, and collagen III. Gene expression is graphed as mean +/− SEM of ΔΔCt compared to unstimulated baseline for n = 4 biological replicates. *p < 0.05. (**C**) Total protein from normal lung fibroblasts or (**D**) IPF lung fibroblasts subjected to western blotting with antibodies to the indicated epitopes. Full-length blots are presented in Supplementary Figures 9 and 10. Quantitative analysis of band intensity from autoradiographs is shown as ratio to GAPDH band intensity, expressed as arbitrary units with unstimulated control at 1, for n = 4 biological replicates. *p < 0.05. Consistent results were seen among all biological replicates.

**Figure 2:**
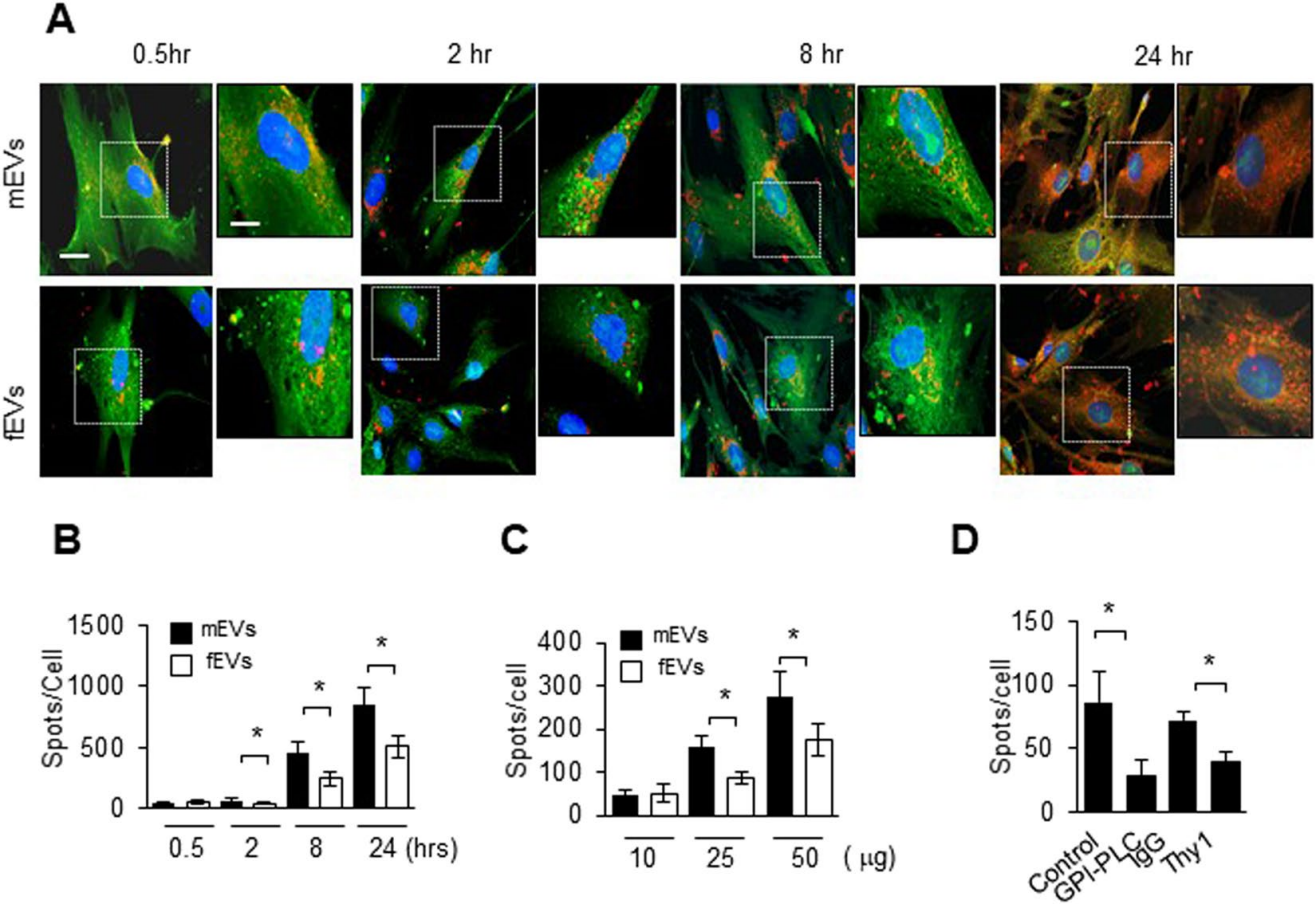
Uptake of mEVs by fibroblasts is Thy-1 dependent (**A**) Representative confocal 3D stack images of EV uptake are shown at various time points (0.5, 2, 8 and 24 hours). Fibroblasts were stained with CFSE (green color) and EVs were stained with CellMask Deep Red dye (red color). 10 μg of EVs were used. Scale bar = 10 μm. The insets show higher magnification (20 × 22 μm rectangle) of the indicated regions of interest. (**B**) IMARIS SPOTS analysis was used to determine the number of EVs per cell in each image shown in (**A**). (n = 3, 9–12 images; 78–80 cells) (**C**) Dose dependent EV uptake (10, 25 and 50 μg) was assessed by 3D confocal imaging at 30 min time point. (**D**) MEVs were pre-treated with phosphatidylinositol-specific phospholipase C (0.1 U/ml PLC) or with anti-Thy-1 (10ug/ml) blocking antibody. 25ug of mEVs were exposed to fibroblasts for 30 mins. Bar graphs show the mean +/− SEM; *p < 0.05.

Normal lung fibroblasts (NLF) (Fig. 1A and C) and fibroblasts derived from lungs of individuals with idiopathic pulmonary fibrosis (IPF) (Fig. 1B and D) were cultured in the presence of TGFβ1, with or without addition of mEVs or fibroblast-derived EVs (fEVs) (10 μg). Both cell types produce EVs of a similar size range (Supplemental Fig. 1A). TGFβ1 stimulation significantly increased mRNA expression of characteristic myofibroblastic molecules, i.e., alpha-smooth muscle actin (α-SMA), EDA-domain containing fibronectin (FN-EDA), type I collagen (Col I), and type III collagen (Col III), in both normal and IPF fibroblasts. MEVs, but not fEVs, inhibited TGFβ1-induced expression of α-SMA and FN-EDA (Fig. 1A and B, upper panels). Normal fibroblasts treated with TGFβ1 and mEVs, or with mEVs pre-incubated with anti-IgG, showed significantly lower levels of α-SMA (51%) and FN1-EDA (52%) in comparison to TGFβ1 treatment alone (Fig. 1A). However, mEVs pre-incubated with anti-Thy-1 antibody had no effect on TGFβ1-induced myofibroblastic differentiation. IPF fibroblasts at basal levels have increased expression of α-SMA, FN-EDA, Col I, and Col III as compared to normal fibroblasts (Supplemental Fig. 2), and mEVs had no significant effect on basal levels of these genes. IPF fibroblasts treated with TGFβ1 in the presence of mEVs or mEVs-α-IgG have significantly lower α-SMA (31%), FN-EDA (54%) and Col III (55%) (Fig. 1B) compared to those treated with TGFβ1 alone, similar to the responses of normal fibroblasts shown in Fig. 2A. Blocking Thy-1 with anti-Thy-1 antibody abrogated the effect of mEVs on alleviating profibrotic responses (Fig. 1A and B). Furthermore, western blotting confirmed the mEV-mediated decreases of α-SMA and FN-EDA expression at the protein level in normal fibroblasts (Fig. 1C) and the decreases of α-SMA and Col III in IPF fibroblasts treated with mEVs or mEVs-α-IgG (Fig. 1D). These effects were also inhibited by Thy-1 blocking antibody (Fig. 1C and D). The basal expression of Thy-1 in either NLF or IPF is similar (Supplemental Fig. 3), and both mEV and fEV express Thy-1 (Supplemental Fig. 1C), although expression is higher in mEV.

### Uptake of mEVs by fibroblasts is Thy-1 dependent

Normal human lung fibroblasts were stained with the intracellular fluorescent dye carboxyfluorescein succinimidyl ester (CFSE). MEVs and fEVs (10 μg) were stained with lipophilic CellMask Deep Red dye, and were incubated with CFSE-stained fibroblasts for a range of time points (Fig. 2A). Adhesion and internalization/uptake of EV was quantified by confocal microscopy as described in Methods. Accordingly, adhesion of mEVs vs. fEVs was detected at 0.5 hr and reached statistically significant differences at 2-hr incubation (Fig. 2B). Incubation of mEV with fibroblasts at 4 °C prohibited mEV uptake (Supplemental Fig. 4). Microscopy findings were confirmed using imaging flow cytometry (Supplemental Fig. 5). Although flow cytometry cannot quantitatively distinguish surface binding and internalization, fluorescence intensity is reduced at 4 °C at all time points, suggesting surface binding but no internalization. Incubation of mEV with fibroblasts at 4 °C had no significant effect on TGFβ1 induced α-SMA and FN-EDA expression (Supplemental Fig. 4). MEVs compared to fEVs demonstrated a 1.9-fold increase in cellular uptake at 8 hours (459 ± 95 vs 246 ± 59 spots/cell) and 1.7-fold increase at 24 hours (847 ± 148 vs 509 ± 86 spots/cell) (Fig. 2B). Concentration-dependent differences in cellular uptake are shown in Fig. 2C. A significant increase of mEV vs. fEV uptake by recipient fibroblasts is demonstrated at 25 μg (159 ± 26 vs 85 ± 15 spots/cell) and 50 μg concentrations (276 ± 56 vs 174 ± 38 spots/cell). Uptake was partially inhibited by removing Thy-1 from EV using phosphoinositide-specific phospholipase C (0.1 U/ml, PI-PLC) or by blocking Thy-1 (10 μg/ml) using anti-Thy-1 antibody (Fig. 2D).

### Thy-1-integin interaction plays an important role in mEV uptake by fibroblasts

**Figure 3:**
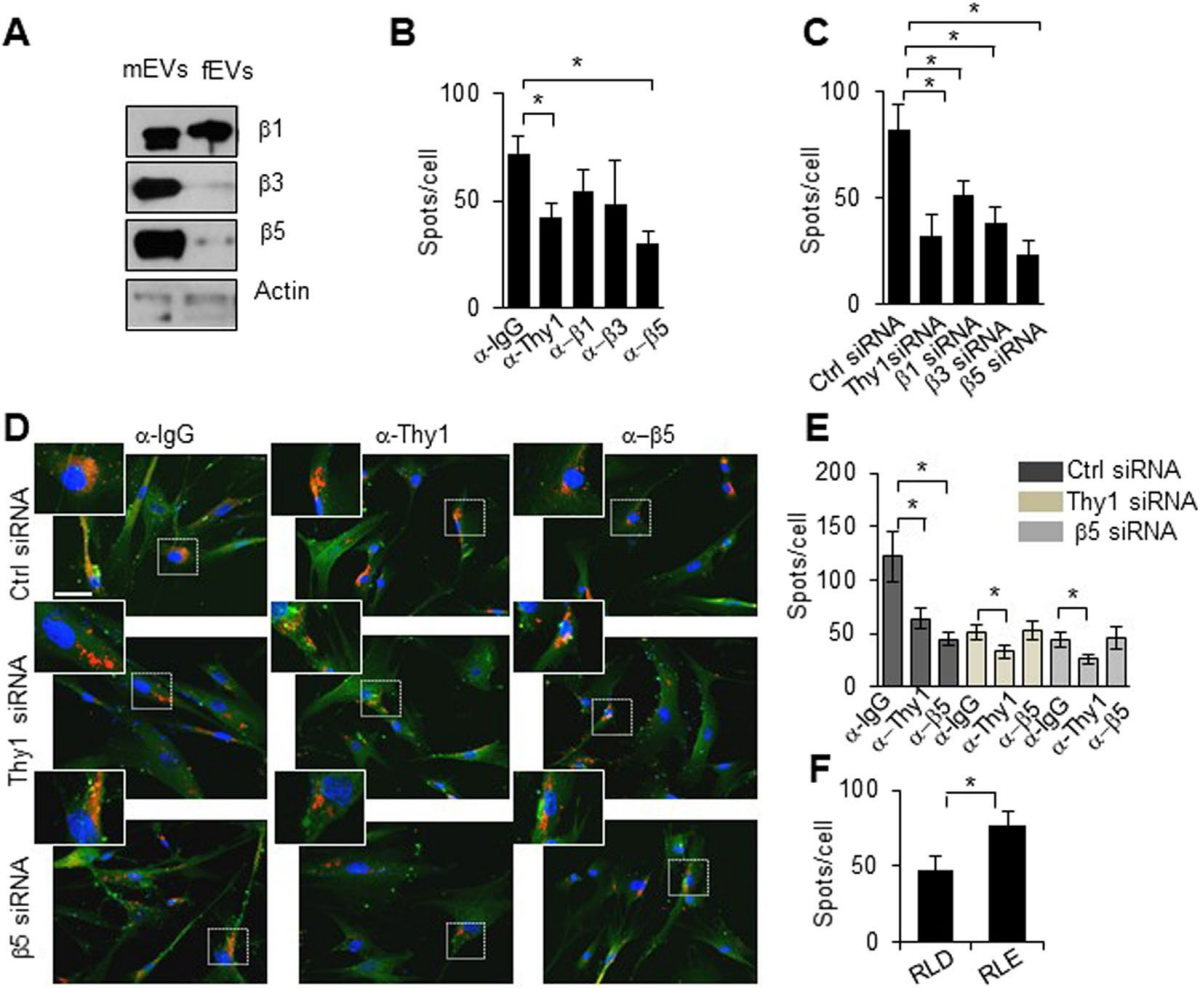
Thy-1-integrin interaction is important in mEV uptake by fibroblasts (**A**) 5 μg of mEVs or fEVs were lysed and assayed by western blotting for the indicated antibodies. Full-length blots are presented in Supplementary Figure 11. (**B**) and (**C**): Statistical analysis of cellular uptake was calculated and is depicted as mean +/− SEM. Blockage of Thy-1, β1, β3, or β5 on mEVs (α-Thy-1, α-β1, a-β3, or a-β5) was done by specific antibody blocking (10 μg/ml). SiRNA was used to downregulate the expression of Thy-1, β1, β3 or β5 in fibroblasts. (**D**) Representative images of EV uptake show that mEVs blocked with anti-Thy-1 or integrin β5 (α-IgG, α-Thy-1, α-β5) antibody co-incubated with fibroblasts treated with scrambled siRNA, Thy-1 or β5 siRNA (Scale bar = 20 μm). The insets show higher magnification (20 × 20 μm square) of the indicated regions of interest. Statistical analysis of (**D**) is shown in (**E**). (**F**) Competitive analysis of mEVs with soluble Thy-1 (1 mg/ml, Thy-1-RLD or Thy-1-RLE) is plotted in the bar graph as mean +/− SEM. 25ug of mEVs were exposed to fibroblasts for 30 mins. (n = 3–4, 9–12 images. *p < 0.05).

Thy-1 is known to interact with β1, β3 and β5 integrins in either *trans* or *cis* ^31^. As shown in Fig. 3A, mEVs have higher expression of integrin β3 and β5, but equivalent β1 expression compared to fEVs. It is also shown (Supplemental Fig. 6) that integrin β1, β3, β5 and Thy-1 are mainly expressed in the smaller exosome fraction. Cellular uptake of mEVs by fibroblasts was decreased by anti-Thy-1 or anti-integrin β5 antibody (Fig. 3B). Antibody to either β1 or β3 also appeared to decrease mEV uptake, but the effect was not statistically significant. To examine the possible roles of cellular Thy-1 and integrin β1, β3, or β5 on mEV uptake, mEVs were co-incubated with fibroblasts in which Thy-1, integrin ββ1, β3, or β5 were knocked down by siRNA (Fig. 3C). The siRNA knockdown efficiency is shown in Supplemental Fig. 7. Cellular expression of Thy-1, β1, β3, or β5 all contributed to cellular uptake (Fig. 3C). To further determine whether blocking Thy-1 or integrin β5 on mEVs together with downregulation of their expression on a cellular level had combinatorial effects on mEV uptake, EVs blocked with control-IgG, anti-Thy-1 or anti-integrin β5 were co-incubated with fibroblasts with control, Thy-1, or integrin β5 siRNA knockdown. There was a small but significant decrease of cellular uptake of mEVs pre-treated with anti-Thy-1 and co-incubated with either Thy-1 or integrin β5 siRNA treated cells (Fig. 3D and E). On the other hand, no additive effect was observed by co-incubating EVs pre-treated with anti-integrin β5 with either Thy-1 or integrin β5 siRNA treated cells. Competition using excess soluble Thy-1, either unmodified or harboring an Asp- > Glu mutation in the integrin-binding domain (RLD to RLE)^29,32^, demonstrated that soluble Thy-1-RLD prevented mEV uptake, but not the RLE mutant, confirming the importance of Thy-1-integrin interaction in mEV uptake (Fig. 3F).

### MicroRNA profiling of mEVs

**Figure 4:**
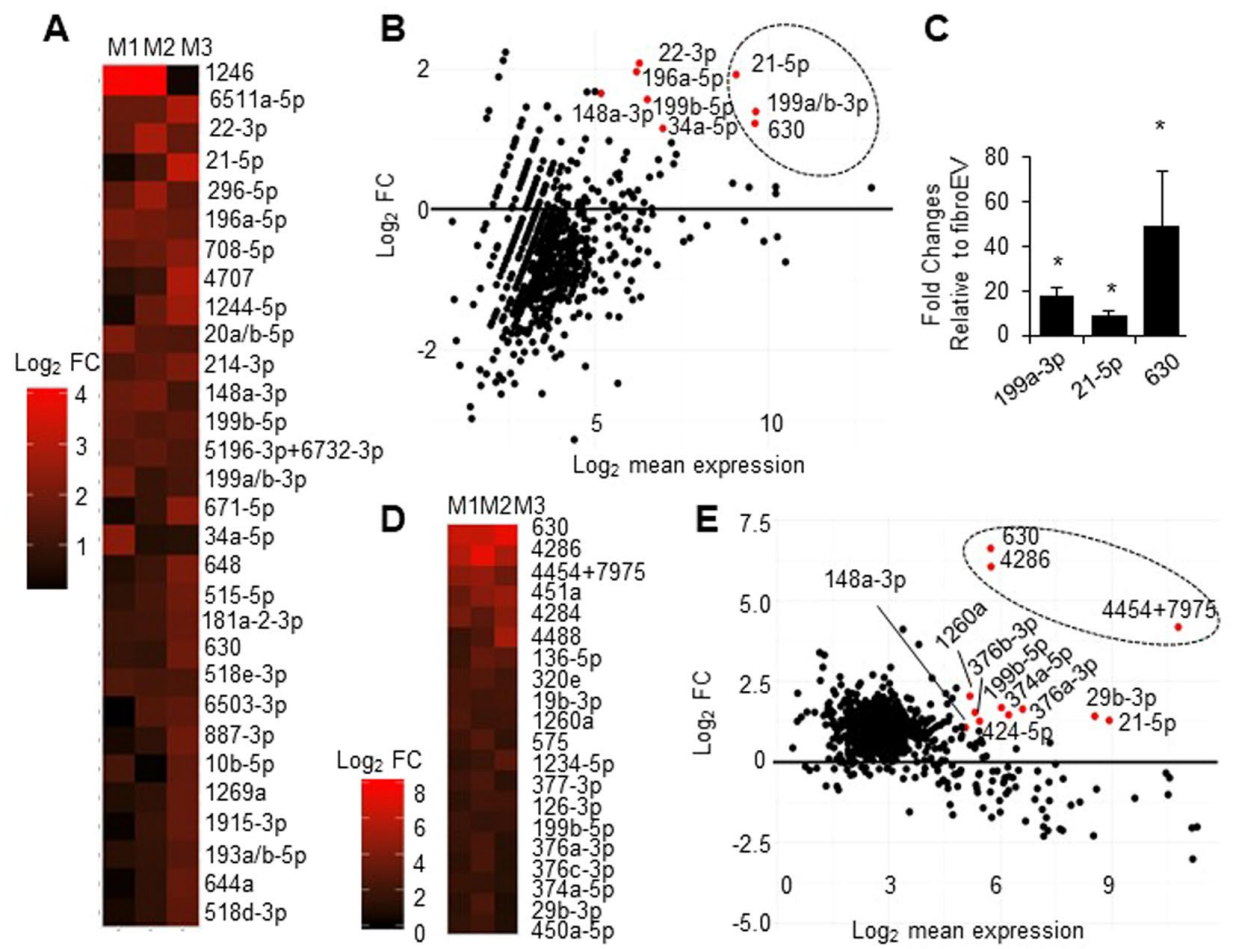
MiR profiling of mEVs compared to fEVs (**A**) Heat map shows the top 30 mEV miRs that differ from fEVs. M1, M2 and M3 were biological triplicates of mEVs from different commercial sources. Log_2_ fold change (FC) indicates fold differences of mEVs vs. fEVs. (**B**) MA plot: Log_2_ FC plotted on the y-axis and Log_2_ mean expression plotted on the x-axis. Red dots indicate the differentially expressed and most abundant miRs in mEVs. (**C**) MiR-21, 199a/b-3p and 630 were experimentally validated by real-time RT-PCR. Black bars show the mean of fold change +/− SEM. (n = 3 *p < 0.05). (**D**) Heat map reveals the top 20 miRs selectively secreted in mEVs compared to their parental cells. (**E**) MA plot shows distribution of miR FC vs expression in mEVs in relative to parental cells. Red dots indicate the differentially packaged and most abundant miRs in mEVs.

MicroRNAs (miRs) can be transferred between cells via EVs^21,33^. We hypothesized that the effect of mEVs on the myofibroblastic phenotype may be mediated through miR transfer. To explore this possibility, the miR content of mEVs from three separate sources/donors was compared to that of fEVs using NanoString analysis, as described in Methods. We identified 30 miRs that were significantly increased in mEVs vs fEVs (Fig. 4A); miR-1246, 6511a-5p, and 22–3p were the top 3 miRs differentially expressed in mEVs (Fig. 4A). MA (log ratio vs. mean average) plot (Fig. 4B) shows the log_2_-fold change (FC) of miR expression in mEVs with respect to fEVs vs log_2_-mean expression in mEV. Red dots in the plot indicate the differentially expressed (y-axis, log_2_ FC > 1) and abundant (x-axis, log_2_ mean expression > 5) miRs in mEVs. MiR-21-5p (log_2_ FC = 1.92, log_2_ mean expression = 9.07), 199a/b-3p (log_2_ FC = 1.39, log_2_ mean expression = 9.64) and 630 (log_2_ FC = 1.22, log_2_ mean expression = 9.65) are the most differentially expressed and also the most abundant miRs in mEVs (Fig. 4A and B). The relative expressions of miR-199a-3p, 21a-5p and 630 in mEVs vs fEVs were further validated by qPCR (Fig. 4C). To identify miRs that were specifically sorted into mEVs, we compared the miR profile from three individual sets of mEVs from different MSC sources/donors vs. their parental MSCs. MiR-630, 4286, and 4454 + 7975 were the top 3 miRs from MSCs sorted into mEVs as shown in the heat map (Fig. 4D). In addition, MA plot shows the differentially packaged and most abundant miRs in mEVs (red dots) with respect to their MSC parent cells. MiR-630 (log_2_ FC = 6.62, log_2_ mean expression = 5.73), 4286 (log_2_ FC = 6.05, log_2_ mean expression = 5.74) and 4454 + 7975 (log_2_ FC = 4.18, log_2_ mean expression = 10.77) are selectively secreted from MSCs (Fig. 4E).

### Molecular targets of mEV miRs in IPF lung fibroblasts

**Figure 5:**
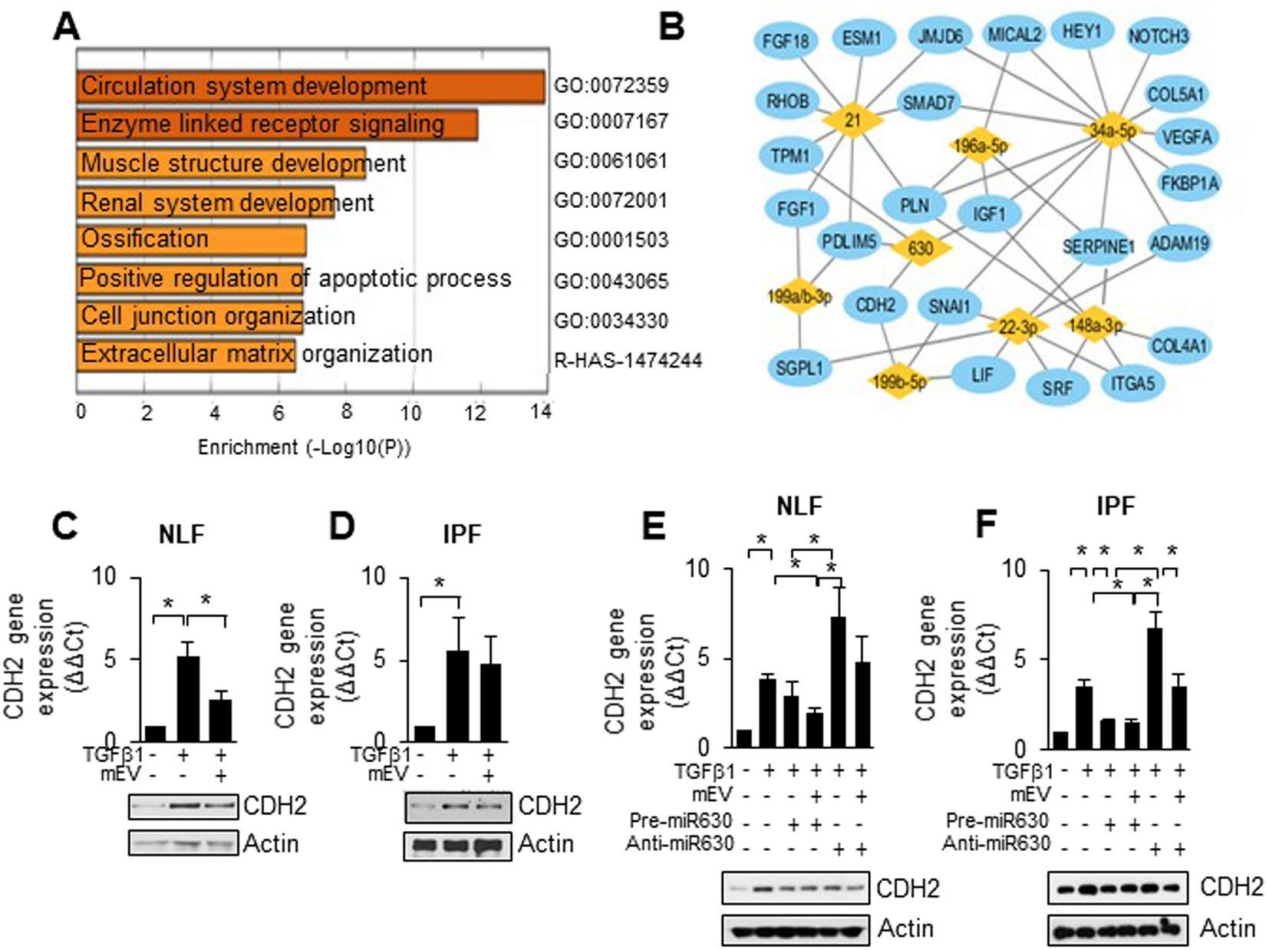
Potential molecular targets of mEV miRs in IPF fibroblasts (**A**) indicates GO biological processes with lowest p-values for genes differentially expressed in IPF vs normal fibroblasts. (**B**) Molecular targets in GO 0072359 are depicted in a miR network based on Gene-microRNAs interactions predicted by miRWalk 2.0 software. (**C**, and **D**) CDH2 gene expression in response mEV treatment was shown by qPCR analysis and western blot. (E) MiR-630 down-regulates TGFβ1-induced CDH2 expression in normal fibroblasts. (**F**) or in IPF fibroblasts (**D**) as shown by qPCR analysis and western blot. Full-length blots are presented in Supplementary Figures 12–15. Bar graphs show the mean +/− SEM of ΔΔCt normalized to control and *p < 0.05 indicated significant differences between control and experimental conditions for n = 4 biological replicates. Consistent results were seen among all biological replicates.

To define possible profibrotic myofibroblast targets of the miRs contained in mEV, we explored *in silico* publicly available transcriptome datasets that indicate differential gene expression of IPF vs. normal fibroblasts. GSE40839^34^. The results showed a clear separation of IPF vs. normal cells by principal component analysis and an increased expression of characteristic myofibroblastic genes α-SMA (*ACTA2*) and *SERPINE1* in IPF fibroblasts, which were selected to be further characterized (Supplemental Fig. 8). The up-regulated genes in IPF fibroblasts in GSE40839 are listed in Supplemental Table 2. Gene ontology (GO) enrichment analyzed by Metascape was used to categorize the up-regulated gene networks in IPF fibroblasts compared to normal lung fibroblasts as shown in Fig. 5A. We used miRWalk 2.0 to filter miR targets clustered in GO term 0072359, and identified 39 genes up-regulated in IPF fibroblasts in GSE40839 (Supplemental Table 3). Several of the miRs enriched in mEVs, including miR-21, 199a/b-3p, 630, 22-3p, 196a-5p, 199b-5p, 34a-5p and 148a-3p, were predicted gene-microRNA pairs with GO: 0072359 genes based on miRWalk 2.0. Gene-microRNA pairs are listed in Table 1 and plotted into networks, as shown in Fig. 5B. Because miR-630 was the top secreted miR from MSCs and not previously identified as modulating fibrotic processes, we chose one of its predicted targets, *CDH2* (N-cadherin), for experimental validation. As shown in Fig. 5C,D,E and F, *CDH2* was induced by TGFβ1 in either normal fibroblasts or IPF fibroblasts and was suppressed by mEVs or by miR-630 mimics. MiR-630 antagonist treatment, on the contrary, enhanced TGFβ1-induced *CDH2* expression. Furthermore, following antagomir-630 treatment, mEVs appeared to be less effective in suppressing TGFβ1-induced CDH2 expression in both normal fibroblasts (Fig. 5E) and IPF fibroblasts (Fig. 5F).

**Table 1:** Gene-microRNA pairs predicted by miRWalk 2.0.

MiRs	Gene-microRNAs pairs
21	ESM1, FGF1, FGF18, JMJD6, PDLIM5, PLN, RHOB, SMAD7, TPM1
199a/b-3p	FGF1, PDLIM5, SGPL1
630	CDH2, IGF1, PDLIM5, TPM1
22–3p	ADAM19, ITGA5, LIF, SERPINE1, SGPL1, SNAI1, SRF
196a-5p	IGF1, MICAL2, PLN, SERPINE1
199b-5p	CDH2, LIF, SNAI1,
34a-5p	ADAM19, COL5A1, FKBP1A, HEY1, IGF1, JMJD6, MICAL2, NOTCH3,PLN, SERPINE1,SMAD7,SNAI1,VEGFA
148a-3p	COL4A1, IGF1, ITGA5, PLN, SEPRINE1, SRF

## Discussion

Extracellular vesicles function in part by delivering nucleic acids and proteins to recipient cells. Many of their nucleic acid-mediated effects require cellular uptake^21,33^. Recent miR studies showed that MSCs overexpressing miR-let7c attenuated renal fibrosis by targeting TGFβR1 expression via exosome uptake^35^. CD34-positive stem cell EVs mediated angiogenic potential in repairing ischemic hindlimbs via delivery of miR-126-3p to endothelial cells^36^. However, the mechanisms for mEV uptake and its antifibrotic effects in lung fibroblasts have not been previously described.

MSC-derived EVs (mEVs) have been shown to mitigate tissue fibrotic responses^20,37^. Recently MSCs have been used clinically in IPF, in a phase I trial^38^. Use of mEVs rather than MSCs may mitigate concerns raised by infusing multipotent cells. In this study, we report that Thy-1 is important in fibroblast uptake of mEVs and thus critical in mEV-mediated inhibition of TGFβ1-induced myofibroblastic differentiation. Thy-1, a GPI-anchored glycoprotein often used as marker of MSCs, is known to interact with integrin via heterotypic (in *trans*) and homotypic (in *cis*) interactions^31,39^. Thy-1-integrin β5 interaction (in *trans*) has been shown to inhibit myofibroblast differentiation^30^. Thy-1-intergrin β3 homotypic (in *cis*) interactions have been shown to mediate fibroblast mechanosensitivity to extracellular matrix stiffness through Src family kinase (Fyn) downstream signaling^29^. Our findings demonstrate that mEV Thy-1 interacts with fibroblasts through integrin β1, β3, or β5 and promotes fibroblast uptake of mEVs. The relative abundance of Thy-1 on mEV (Supplemental Fig. 1C) supports its functional importance. Based on our data it is also likely that fibroblast cell surface Thy-1 interacts with β5, and possibly also β1 and β3, integrins on mEV. In pathologic fibroblastic foci, although we have demonstrated little or no expression of Thy-1^40^, interaction between mEV Thy-1 and cellular expressed integrin (in *trans*) could compensate for the Thy-1 deficiency in the foci and thus promote mEV uptake. The described overexpression of integrin αvβ5 in fibroblastic foci^41^ could attract Thy-1 rich mEV to the lesional myofibroblasts. RLD, the Thy-1-integrin-binding motif, is critical in mEV-fibroblast interaction. MEV uptake can involve mEV Thy-1 or cellular Thy-1 to engage signaling. As shown in the recent study by *Li et al*., cellular Thy-1 is also critical in human cytomegalovirus (HCMV) entry^42^. The interaction between cellular Thy-1 and HCMV gB or gH may constitute a molecular complex important for HCMV entry^31^. Because virus particles are similar in size to EVs and share similar biogenesis pathways^43^, mEV entry into fibroblasts may utilize a similar Thy-1-dependent pathway to facilitate EV-cell communication and delivery of EV contents.

The presence of Thy-1 on the EVs could indicate that lipid raft associated activity contributes to releasing exosomes from mutlivesicular endosomes. As suggested by Gassart *et al*.^44^, lipid rafts could serve a weak point on the membrane surface, promoting bending or budding. The concentration of lipid rafts in exosomes not only provides lateral aggregation with cholesterol/phospholipids, but also supports lipid-protein and protein-protein interactions. Investigation of sorting from plasma membrane to exosomes will further shed an insight on mEV secretion and its role on intercellular signaling.

TGFβ1-induced myofibroblastic differentiation is a critical factor in the pathogenesis of idiopathic pulmonary fibrosis (IPF)^45^. We showed that mEVs alleviated TGFβ1-induced α-SMA expression in lung fibroblasts and also decreased the expressions of fibronectin and collagen III. These results are consistent with a recent report showing alleviation of TGFβ1-induced α-SMA expression in dermal fibroblasts by umbilical cord MSC-derived EVs^46^. We further demonstrated that incubation at 4 °C or blocking Thy-1 on mEVs reduced mEV uptake and inhibited mEV antifibrotic effects, suggesting that the Thy-1 mediated mEV uptake is critical to mitigate TGFβ1 effects. Once mEVs are internalized, functional miRs could target effectors of myofibroblast differentiation. It has been shown that miR-21, together with miR-23, 125, and 145 derived from umbilical cord MSCs, can target SMAD 2, TGFβ2 and TGFβR2, and thus downregulate TGFβ1 signaling^46^. In the current study, we explored the likelihood that miRs enriched in mEVs; such as 199a-3p, 21-5p, 630, 22-3p, 196-5p, 199b-5p, 34a-5p and 148a-3p, contribute to the inhibition of myofibroblast differentiation. Some of these miRs have already been demonstrated to have either pro- or anti-fibrotic roles: serum miR-21 in EV correlates with poor prognosis in IPF^47^, and miR-21 activates myofibroblasts *in vitro* ^48^; miR-199 is implicated in liver fibrosis^49^, and is also upregulated in IPF and activates myofibroblasts^50^; miR-22 suppresses cardiac fibrogenesis^51^ and cirrhosis^52^; miR-196-5p mitigates renal fibrosis^53^; and miR-34-5p is profibrogenic in the heart^54^ and regulates pneumocyte senescence in IPF^55^. MiR-630, the most highly enriched miR in mEVs in our study, has been described in another study on MSC-derived EVs^20^, but has not been directly implicated as regulating fibrosis. MiR-630 can specifically target human *SNAI2* (Snail 2) in suppressing epithelial to mesenchymal transition (EMT) in lung and liver cancer cells^56,57^; however, its role in myofibroblast differentiation has not been previously shown. From its downstream target, *CDH2*, miR-630 may regulate adherens-junction dependent cell migration^58^ and fibroblast invasion^59,60^. Moreover, *CDH2* could affect fibroblast mechanotransduction^61^. The β-catenin signaling pathway downstream of *CDH2* has substantial crosstalk with TGFβ1^62,63^ and integrin signaling^64,65^ in the regulation of myofibroblast differentiation and function. Further investigation is required to better define the roles of miR-630 in modulating myofibroblast differentiation. However, because multiple miRs co-exist in mEVs, the impact of a single miR may not be as important as a cluster of miRs. The common predicted molecular targets shown in Fig. 5B regulated by multiple miRs suggest a multimodal effect of mEV on the suppression of IPF phenotypes. Insulin-like growth factor 1 (IGF1) and serpin family E member 1 (SERPINE1), well-known factors in promoting fibrotic development can potentially regulated by miR-196a-5p, 34a-5p and 148a-3p. MiR-196a-5p and 34a-5p have been reported to inhibit fibrosis through other downstream effectors as well. Examples are TGFβR2 targeted by miR-196a-5p in renal fibrosis induced by unilateral ureteral obstruction^53^ and sirutin 1, cyclin E2, cyclin D1 and E2F3 targeted by miR-34a-5p in bleomycin-induced lung fibrosis^66^. Thus, mEV may target multiple pathways simultaneously in mitigating fibrosis. Taken together, our findings show Thy-1-mediated mEV uptake and anti-myofibroblastic effects in IPF fibroblasts, and reveal miRs enriched in mEV that may mediate these effects. Further investigations will elucidate in greater detail the mechanisms by which mEV miRs modulate myofibroblast differentiation.

## Methods

### Extracellular vesicle (EV) isolation

Human bone marrow-derived MSCs and culture medium were purchased from RoosterBio Inc. (Frederick, MD) and Lonza (Walkersville, MD). MSCs were cultured in low percentage of fetal bovine serum and were used between passages 2-5 using proprietary RoosterBio culture media. Human lung fibroblasts (CCL-210, ATCC, Manassas, VA) were grown in DMEM with 10% FBS). Serum was pre-ultracentrifuged (100,000 × *g*) 18 hours to deplete existing extracellular vesicles. Conditioned media were collected every 2–3 days and stored at −80 °C until the accumulation of 300 ml. Cellular debris was removed by low-speed centrifugation at 300 × *g* for 30 minutes. Microparticles (500–1000 nm) were pelleted at 10,000 × *g* for 20 minutes. EVs (50–500 nm) were pelleted by ultracentrifugation at 100,000 × *g* for an hour in an SW32i swinging rotor centrifuge (Beckman Coulter, Indianapolis, IN). EVs were then further washed in 25 ml once with PBS and re-centrifuged at 100,000 × *g* for an hour, after which the supernatant was removed and the final EVs were re-suspended in 400 μl PBS for immediate use or stored at −80 °C. The protein concentration of EVs was measured using the BCA protein assay kit (Thermo Fisher, San Diego, CA).

### Transmission electron microscopy (TEM)

EV pellets were fixed in 4% paraformaldehyde and pre-embedded with agarose. 1-mm^3^ cell blocks were mounted onto specimen holders and snap frozen in liquid nitrogen. 80 to 90 nm frozen sections were picked up with a 1:1 mixture of 2.3 M sucrose and 2% methylcellulose (15 cP) and transferred onto Formvar and carbon-coated copper grids. Sectioned slices were blocked with using 1% cold water fish-skin gelatin and incubated with primary antibody (anti-hThy-1: 1:200; anti-CD63: 1:50, see antibody sources below, in 1% BSA/PBS) for 1 h. Coverslips were washed three times in PBS (for 15 min each), incubated with secondary antibody (anti-mouse IgG-12nm gold and anti-rabbit IgG-18nm gold), washed three times with PBS (for 15 min each time). Grids were viewed using a JEOL 1200EX II (JEOL, Peabody, MA) transmission electron microscope and photographed using a Gatan digital camera (Gatan, Pleasanton, CA), or viewed using a Tecnai G2 Spirit BioTWIN transmission electron microscope equipped with an Eagle 4 K HS digital camera (FEI, Hillsboro, OR).

### Nanoparticle tracking analysis (NTA)

EV pellets in PBS were first normalized to protein content and then subject to NTA analysis. NanoSight NS-300 (Malvern, Worcestershire, UK.) equipped with 405 nm laser. Background noise was eliminated by adjusting exposure time. Briefly, three independent videos of 60 s intervals were taken and analyzed by NTA software (Nanosight 2.1).

### EV labeling and Immunofluorescence

Freshly isolated or freeze-thawed EVs were resuspended in 400 μl of PBS at 0.1–0.2 μg concentrations. EVs were stained with CellMask Deep Red with excitation/emission at 649/666 nm (Thermo Fisher Scientific). For labeling, EVs were incubated with Deep Red dye (1:1000) for 20 mins at 37 °C. The unincorporated dyes were removed by extensive PBS washing (1 to 10,000 v/v ratio) and EVs then were pelleted down at 100,000 × *g* for one hour. The EV pellet was diluted in PBS and protein concentration measured by BCA protein assay kit. Cells were stained with CellTrace™ Carboxyfluorescein succinimidyl ester (CFSE, Life Technologies, Carlsbad, CA) which has excitation/emission maxima at 492/517 nm. CFSE dyes can diffuse into cells and bind covalently to intracellular amines upon digestion by intracellular esterases, forming stable fluorescent staining. Due to this covalent coupling reaction, fluorescent CFSE can be retained within the cell and not transferred to adjacent cells giving the defined cytoplasmic space. 3–5 × 10^5^ cells in the serum free media were stained with CFSE dye at 1:1000 dilution (working concentration at 5 μM). Incubation was carried out at 37 °C for 20 mins protected from light. The solution was pelleted and washed with serum free medium at 1:10 ratio to remove the free dye. Cells were then subcultured to the 8-well chamber slides (Millipore, Billerica, MA). CFSE stained cells were incubated with EV in various time points and treatment. Cells were washed and fixed using 3.7% (w/v) formaldehyde for 5 min at room temperature and were prepared for cellular imaging. To eliminate non-specific membrane dye transfer, 4 °C control experiments allowed set up to obtain the passive dye diffusion at the 2 hour incubation compared to 37 °C condition (Supplemental Figs 4 and 5). An Olympus FV1000 confocal laser-scanning microscope was used to acquire 3D-stacking images using a 40X/1.2 NA oil-immersion lens at an acquisition resolution of 1024 × 1024 in 8 μm per second. Pinhole diameters were set to less than 1 airy unit and optical slice sections of 0.55 µm were taken. Image J volume viewer was used to process 3D-stacking images in maximal density of Z-projection. Quantification of EV uptake was analyzed using SPOTS module of the IMARIS software package (Bitplane AG, Switzerland) on a per pixel basis^67^. Briefly, SPOTS analysis was carried out by the selection of Deep Red + spherical-like particles with a minimal 500 nm diameter (~5 pixels), which is modified from the original reference^67^ which used 2 μm spot selection to indicate the extracellular vesicles. We performed a more restrictive spot selection of 500 nm diameter based on our nanoparticle tracking data (Supplemental Fig. 1A). Intracellular particles were those coincident with CFSE (i.e., intracellular). Detected spots were further filtered through a quality control step (10–15% of overall intensity set as threshold values).

### Reagents and Western blotting

TGFβ1 was purchased from Peprotech (Rocky Hill, NJ). The primary antibodies were used at 1:1000 ratio. Monoclonal anti-CD63 (H5C6, The Developmental Studies Hybridoma Bank, Iowa City, IA), anti-CD81 (MAB 4615, R&D Systems, Minneapolis, MN), anti-Thy-1.2 (HO-13-4, ATCC and 550402, BD Biosciences, San Diego, CA), anti-GAPDH (GTX627408, GeneTex, Irvine, CA) and rabbit polyclonal anti-calnexin (2679S, Cell Signaling, Danvers, MA) were used to detect EV and cellular proteins. Monoclonal anti-FN-EDA (ab6328, Abcam, Cambridge, MA), monoclonal anti-collagen I (GTX26308, GeneTex) and rabbit polyclonal anti-α-SMA (CBL171-I, EMD Millipore, Temecula, CA) anti-collagen III (GTX111643, GeneTex) and anti-N-cadherin (GTX127345, GeneTex) were used in western blotting studies.

### Isolation of RNA and small RNA analysis

Total RNA was isolated from EVs, MSCs and fibroblasts using miRCURY RNA Isolation Kit (Exiqon, Woburn, MA). EV pellets or cells were immediately lysed by lysing solution and processed according to the manufacturer’s protocol. Cellular and EV total RNA concentration was determined using NanoDrop.

### NanoString miRNA array and bioinformatics analysis

For the NanoString miRNA array (nCounter Human v3 miRNA Expression Assay, NanoString, Seattle, WA), 100 ng of RNA extracted from EVs or cells were used as a starting material. Briefly, the miRs were ligated to a species-specific tag sequence (miRtag) via ligation and hybridized subsequently. The normalization factor was generated using the geometric mean of the top 100 miRs for each sample and analyzed by nSolver software^68^. The normalization results were then imported in R/Bioconductor to generate heatmaps and MA plots. MA plot is a plot of log_2_ (fold change) versus log_2_ (mean expression). Predicted gene-microRNA pairs were obtained from the miRWalk 2.0 online database^69^ and visualized by Cytoscape version 3.4^70^. Biological process gene ontology was processed using Metascape.

### Quantitative PCR for profiling gene expression and mature miR expression

Total RNAs were isolated by TriPure reagent (Roche Life Science, Indianapolis, IN) and cDNA were synthesized using Takara RT scripts (Takara Bio USA, Mountain View, CA). Quantitative PCR was performed using the Bio-Rad iCycler iQ5 (BioRad, Hercules, CA). The sequence of cDNA primers for a-SMA, Col I, Col III, FN1-EDA, N-cadherin and GAPDH are listed in supplemental Table 1. Relative changes in expression were determined by normalization to GAPDH (Ct value). Comparative threshold (ΔΔCt) was calculated between different experimental conditions. Mature miR primers (miScript primer assay) were purchased from Qiagen (Qiagen, Frederick, MD).

### Flow and imaging cytometry

CCL-210 cells were cultured to 80–90% confluence. The day before EV-adhesion, cells were washed 2x with 0.1 μm filtered PBS and the media replaced with exosome-free FBS media (exo-free CM). The day of EV adhesion, CCL-210 were trypsinized and resuspended in 100 μl 0.1 μm filtered fluorobrite DMEM (4 conditions: EVs at 4 °C; EV-Free medium at 4 °C; EVs at room temperature (RT); EV-Free medium at RT). CellMask Deep Red was used to label EVs (a 1:1000 dilution of commercial stock). EVs were incubated at RT for 0.5 hrs and then washed with 1 ml of filtered PBS and centrifuged at 50,000 × *g* for 1hr. EVs were washed one more time with PBS and the EV pellet resuspended in 50 μl. EVs were left out at RT overnight for dye to leach. Next day, EVs were centrifuged 30 mins at 50,000 × *g*. Pellet was resuspended and counted for EVs. Supernatant (EV-Free) was centrifuged 2 more times for 30 mins at 50,000 × *g* to remove any residual EVs. EVs in EV-Free supernatant were counted. EV enumeration was performed on ImageStreamX MkII without Brightfield or SSc. 20 × 10^6^ EVs or equal volume of EV-Free supernatant were added to CCL-210 cells and allowed to adhere for 30, 60 and 120 minutes before cells were collected on ImageStreamX MkII. Cells were also collected prior to EV addition at time 0.

### Statistical analysis

One-way ANOVA followed by Tukey-Kramer test for unequal sample sizes was used to compare multiple groups using GraphPad Prism 6.0. For non-normally distributed data, differences between two groups were determined using the Mann-Whitney U test for unpaired observations. Variables are reported as mean ± SEM. A p value of < 0.05 was considered statistically significant.

## Electronic supplementary material

Supplementary Information
