# Supplementary material for: Thy-1 dependent uptake of mesenchymal stem cell-derived extracellular vesicles blocks myofibroblastic differentiation
Source: Sci Rep. 2017 Dec 22;7:18052. doi: 10.1038/s41598-017-18288-9 (PMC5741716; doi:10.1038/s41598-017-18288-9)
Supplement: Supplementary file 1 — Supplementary Information [file 41598_2017_18288_MOESM1_ESM.pdf]

Supplementary Information file

Thy1-dependent uptake of mesenchymal stem cell-derived extracellular vesicles blocks myofibroblastic differentiation.

Tzu-Pin Shentu*, Tse-Shun Huang, Mateja Cernelc-Kohan, Joy Chan, Simon S Wong, Celia R Espinoza, Chunting Tan, Irene Gramaglia, Henri van der Heyde, Shu Chien and James S Hagood*.

*: To whom correspondence should be addressed:

James S. Hagood, M.D.
jhagood@ucsd.edu

Tzu-Pin Shentu, PhD.
tpshentu@gmail.com

Supplemental Figure 1

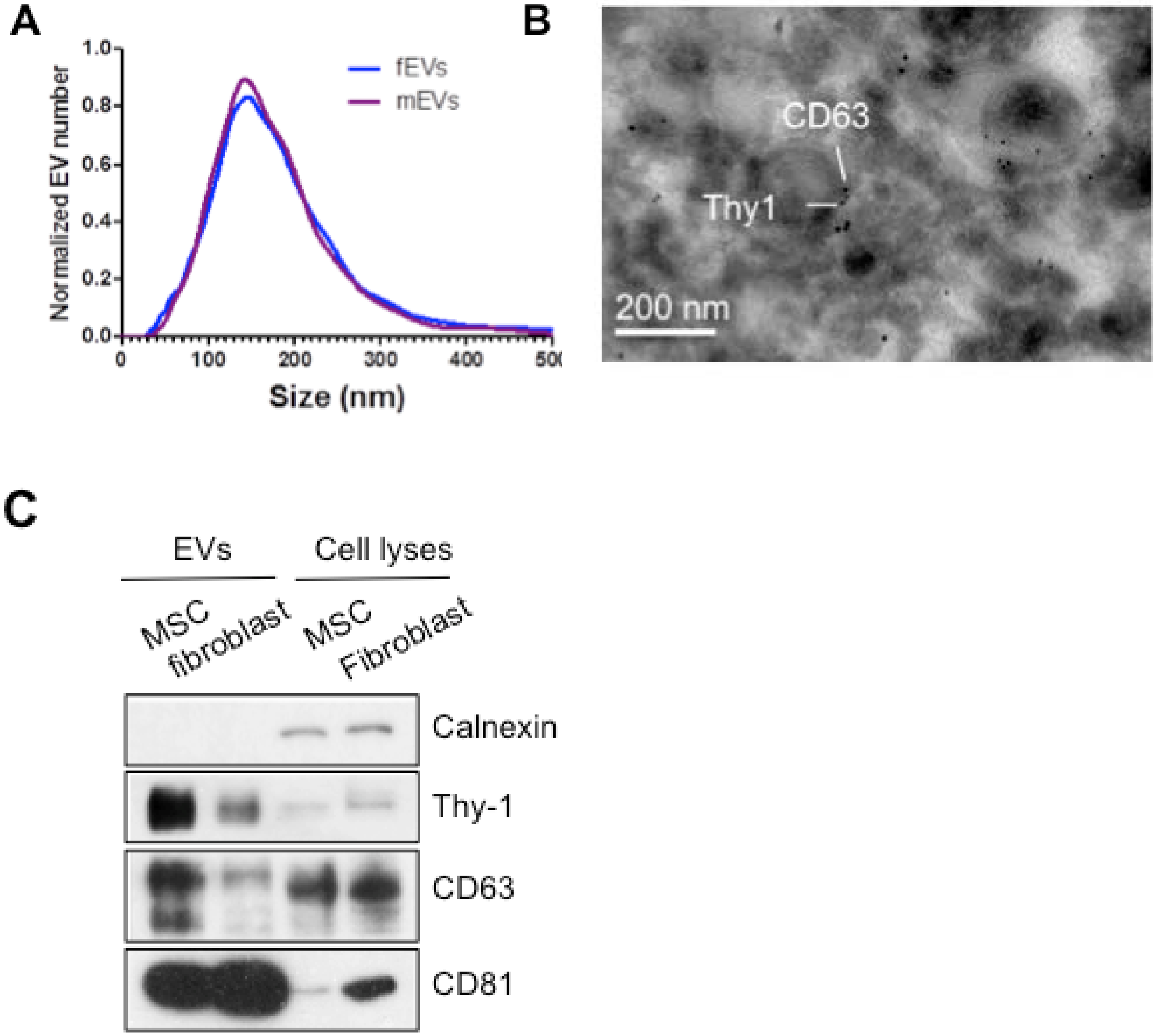

Supplemental Figure 1. Thy-1 expressed on hMSC- and fibroblast-derived extracellular vesicles (mEVs). (A) Nanoparticle tracking analysis shows similar size distribution of extracellular vesicles (EVs) released from MSC and fibroblasts. Average sizes of EVs are 152± 1.8 and 160±2.8 nm isolated from MSCs and normal lung fibroblasts respectively. (B) mEVs were stained with antibodies to Thy-1(12nm gold beads) and CD63 (18nm gold beads) and visualized by electron microscopy ; scale bar is 200nm. (C) 5μg of EVs and 20μg of cellular lysates were assayed by western blotting for the indicated antibodies. Both mEV and fEV contain EV makers CD63 and CD81 and are free of calnexin.

Supplemental Figure 2

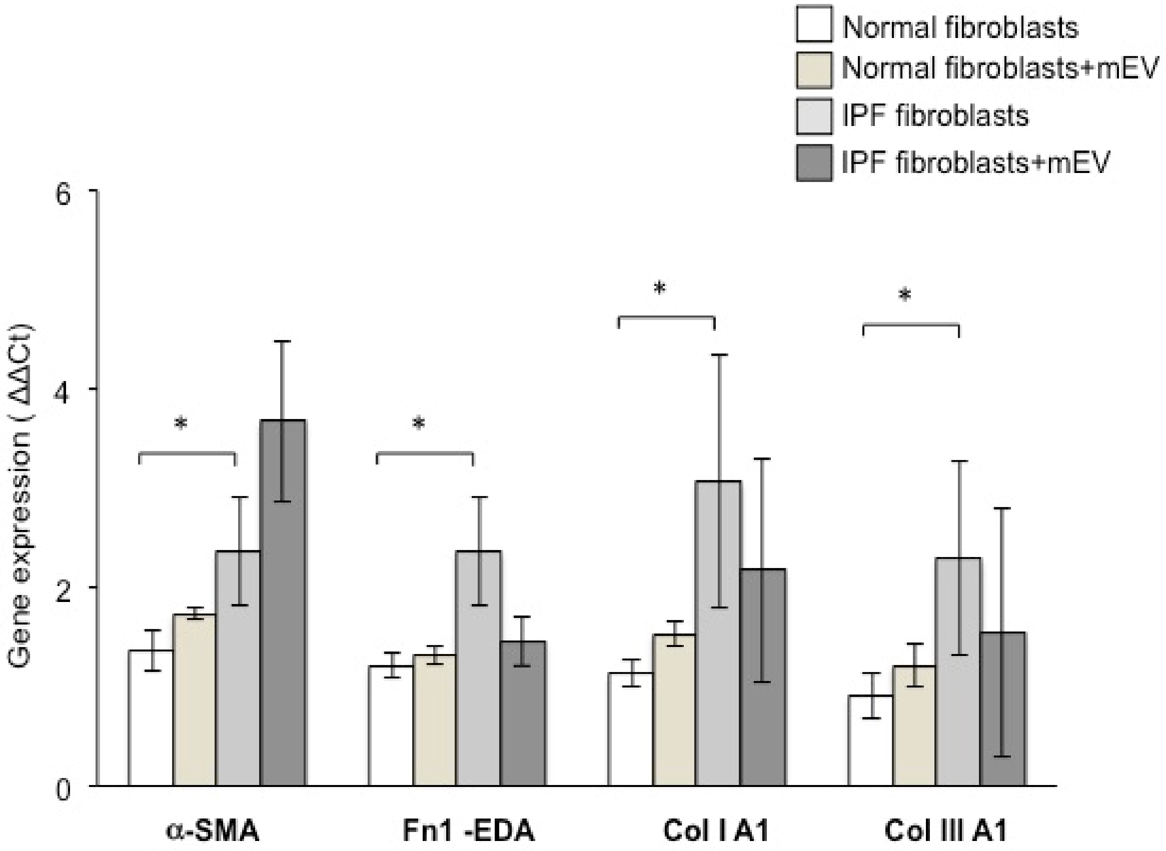

Supplemental Figure 2. Basal gene expression of a-SMA, fibronectin-EDA, collagen A1, and collagen III A1 among normal fibroblasts (NLF), NLF treated with mEV, IPF fibroblasts and IPF fibroblasts treated with mEV. Total RNA was subjected to RT-PCR using primers for human α-SMA, FN1-EDA, collagen I, and collagen III. Gene expression is graphed as mean +/- SEM of ΔΔCt compared to normal fibroblast at baseline 1 for n = 3-5 biological replicates. *: $p < 0.05$.

Supplemental Figure 3

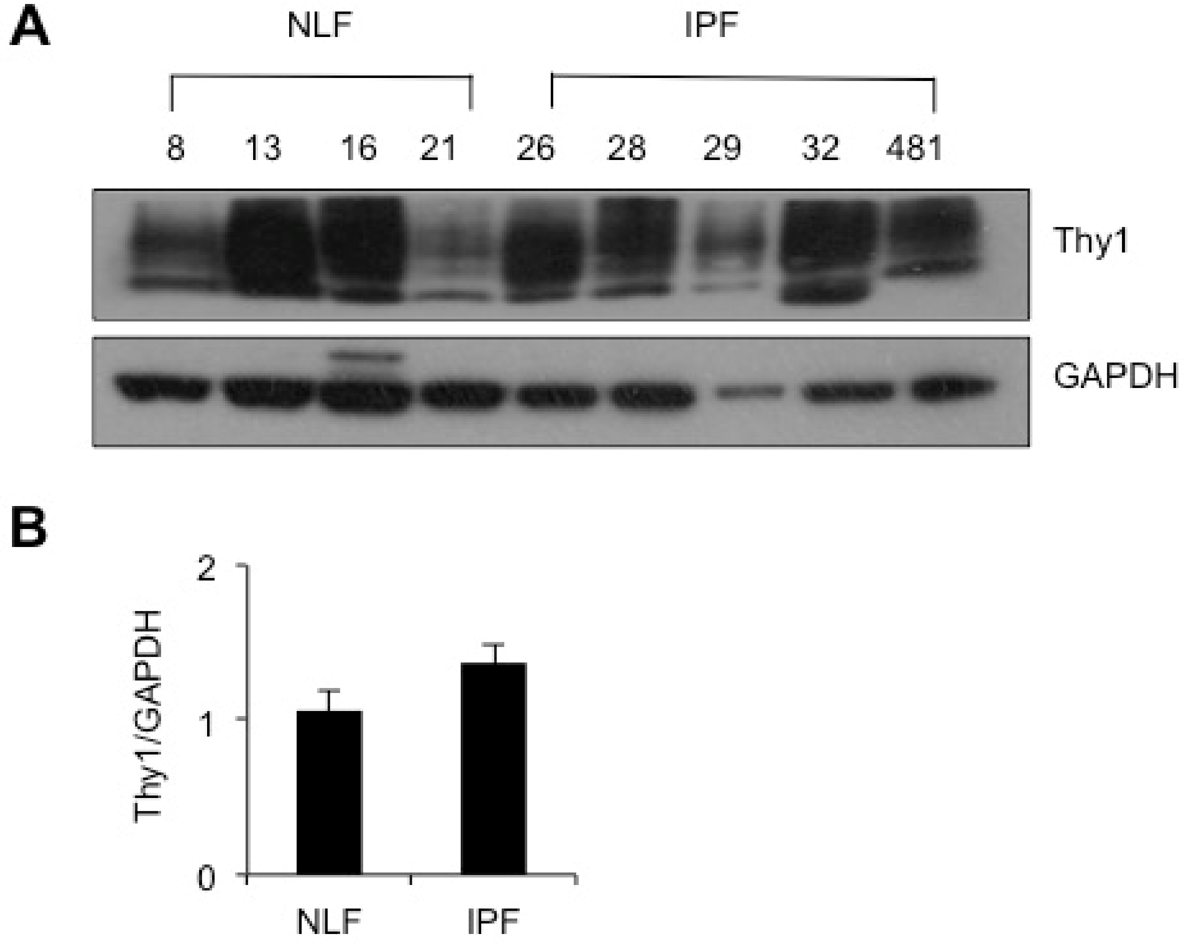

Supplemental Figure 3. Thy-1 expression in normal lung fibroblasts and IPF fibroblasts (A) Representative western blot shows Thy-1 expression among different normal lung fibroblasts (NLF) and IPF lung fibroblasts (IPF). (B) Statistical analysis of Thy-1 expression (band intensity mean +/- S.D.) in (A) using Image J.

Supplemental Figure 4

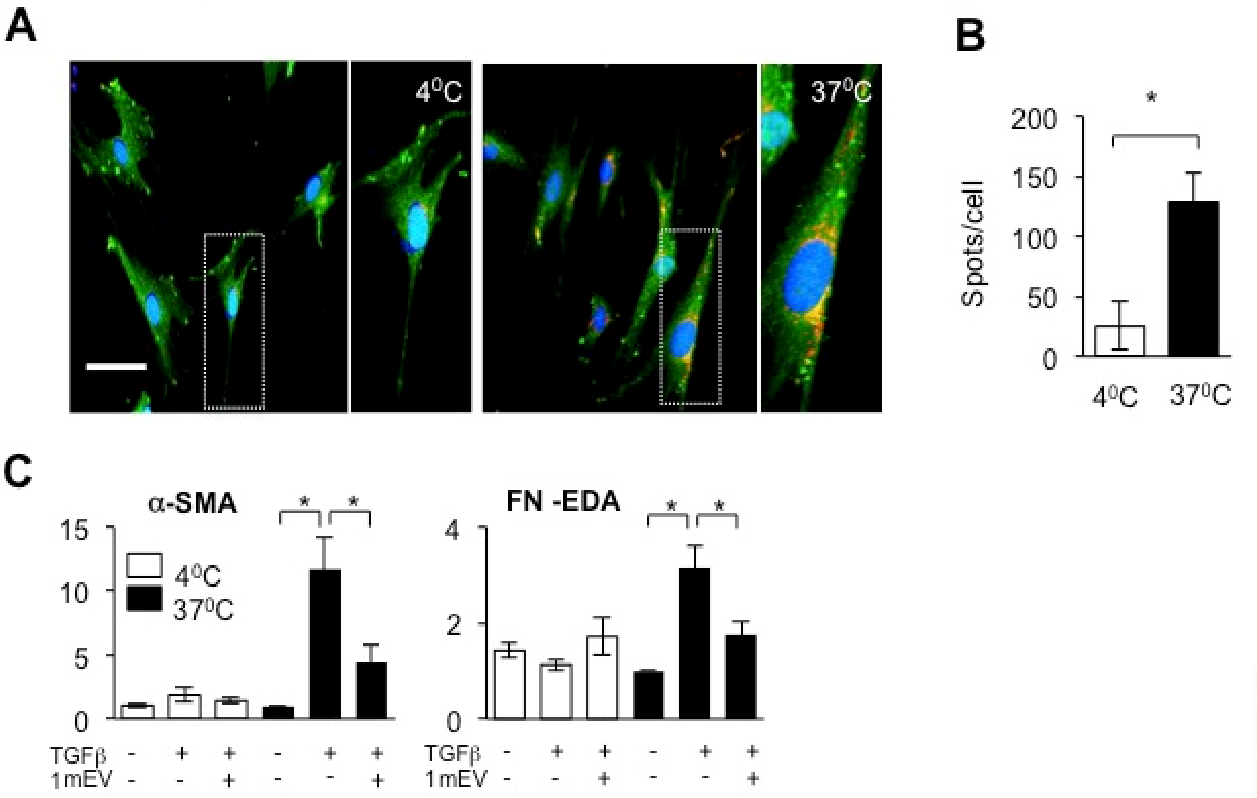

Supplemental Figure 4. EVs uptake at 4°C compared to 37°C (A) Representative confocal 3D stack images of EV uptake at 4°C compared to 37°C at 2 hour incubation. Fibroblasts were stained with CFSE (green color) and EVs were stained with CellMask Deep Red dye (red color). 10 μg of EVs were used. Scale bar=20 μm. The insets show higher magnification (20x60 mm rectangle) of the indicated regions of interest. (B) IMARIS spot analysis was used to determine the number of EVs per cell in each image shown in (A). (n=2, 10 images; 43-57 cells) (C) Total RNA at 4°C or 37°C was subjected to RT-PCR using primers for human α-SMA and FN1-EDA. Gene expression is graphed as mean +/- SEM of ΔΔCt normalized to normal fibroblast incubated at 37°C (arbitrary units) for n = 3 biological replicates. *: $p < 0.05$.

Supplemental Figure 5

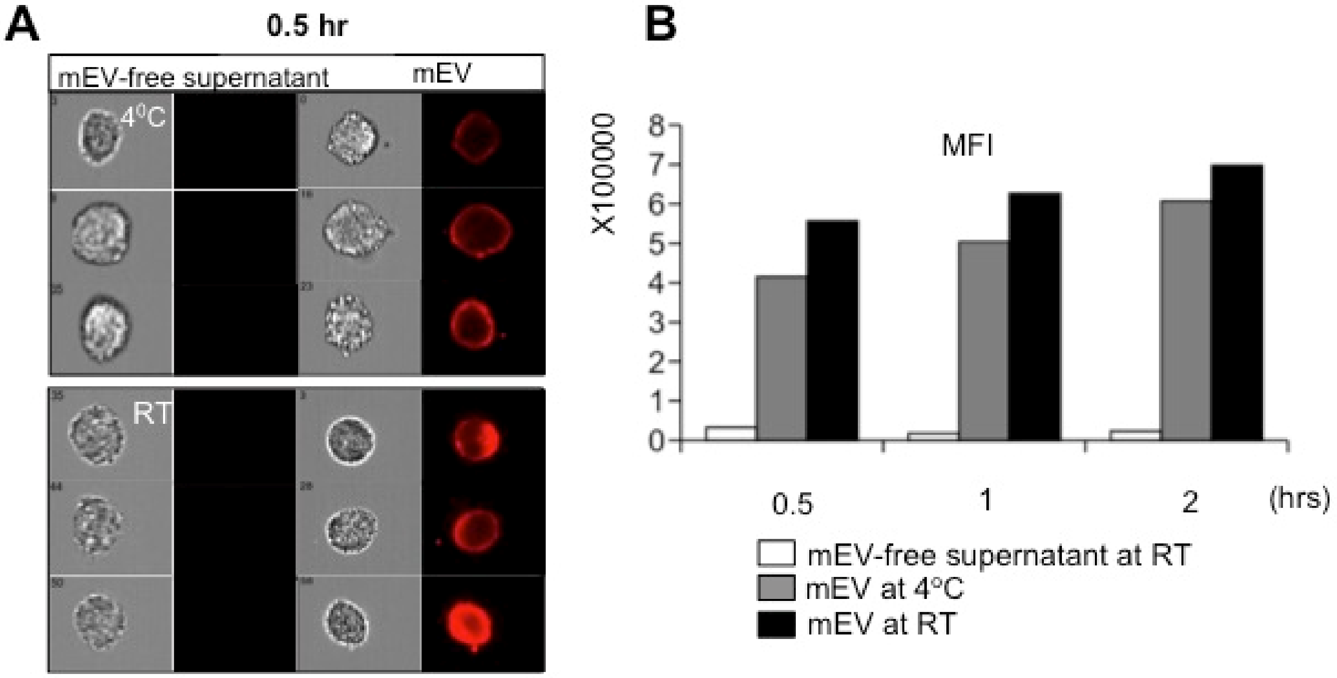

Supplemental Figure 5. Flow cytometry measurement of mEVs uptake at 4ºC compared to room temperature (RT) (A) Representative flow cytometry images of mEV uptake at RT compared to 4ºC at 0.5 hour incubation. mEVs were stained with CellMask Deep Red dye (red color). (B) Mean fluorescent intensity analysis of images in (A) at over time.

Supplemental Figure 6

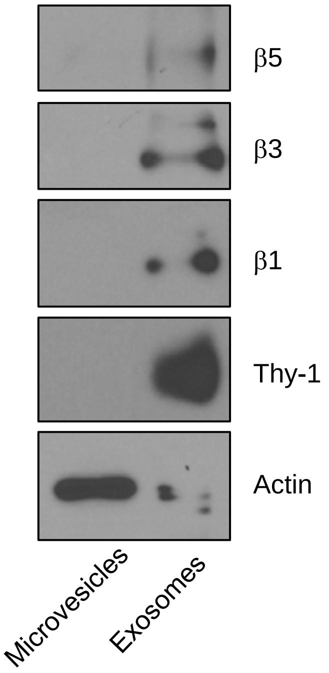

Supplemental Figure 6. Immunoblot of MSC-derived Thy-1, integrin β1, β3, or β5 in the exosome fraction vs. microvesicle fraction. 5mg of exosomes or microvesicles were loaded.

Supplemental Figure 7

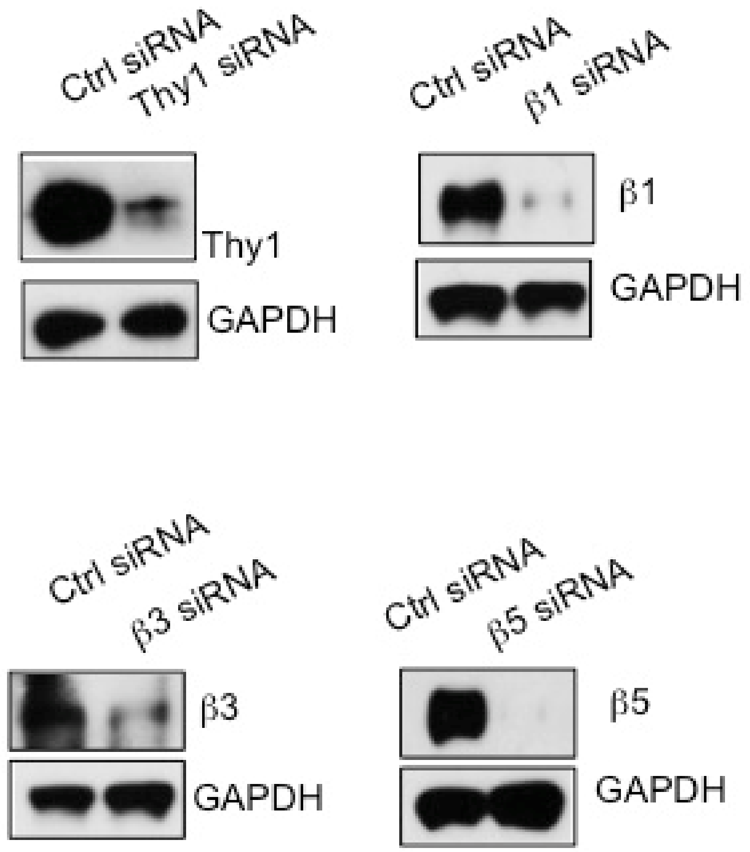

Supplemental Figure 7. Western blot of siRNA knockdown of Thy1, integrin β1, β3, β5 in normal fibroblasts. Thy1, β1, β3 and β5 siRNAs were used to knock down cellular expression of Thy1, β1, β3 and β5. GAPDH was used as the internal control**.**

Supplemental Figure 8

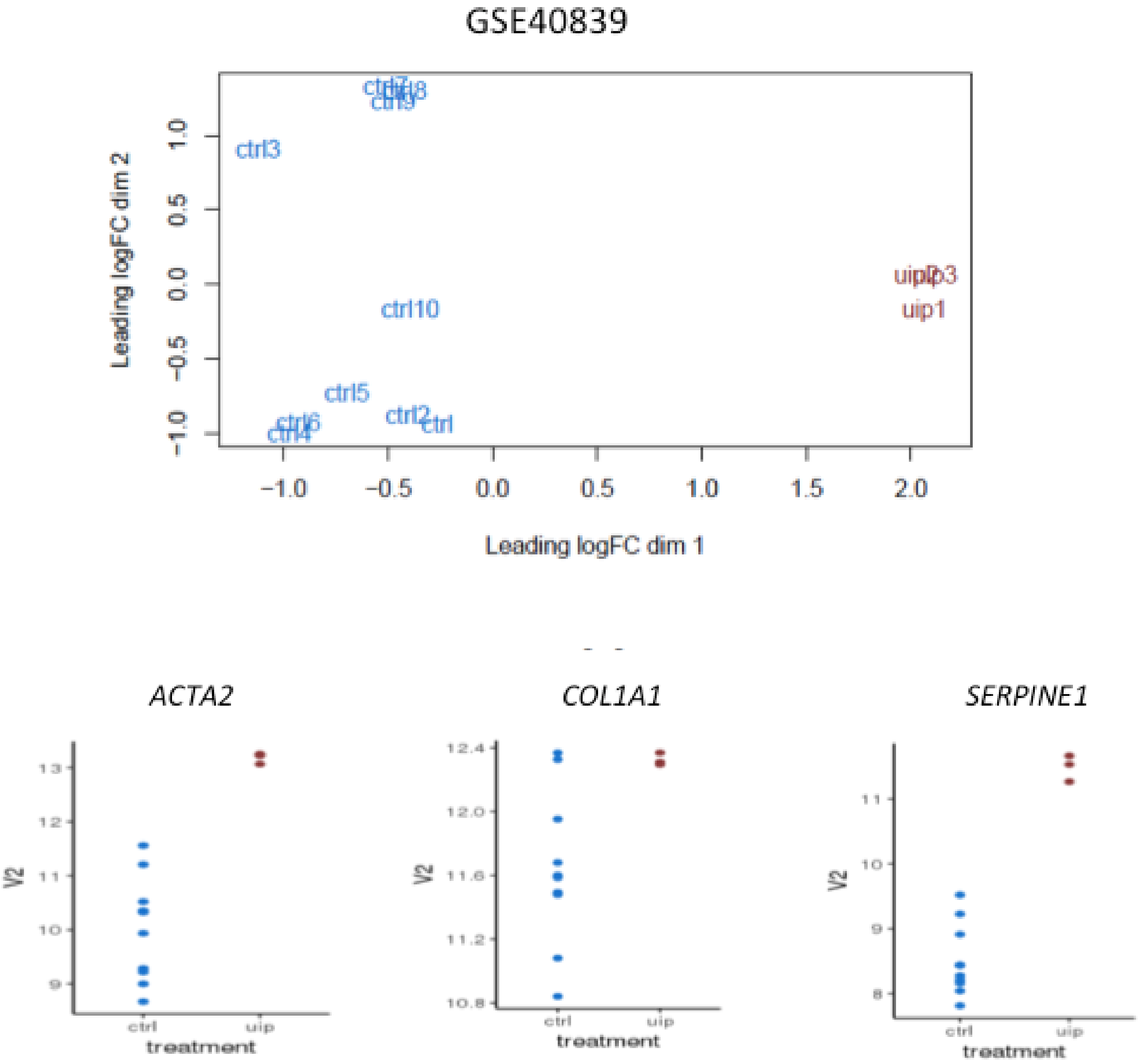

Supplemental Figure 8. Differential expression of myofibroblastic genes in the GSE 40839 dataset. (A) PCA plot with whole expressed transcriptomes of GSE40839. (B) Normalized RNA expression levels of genes that are known to be up-regulated in IPF myofibroblasts.

Supplemental Figure 9: Full western blot of Figure 1C

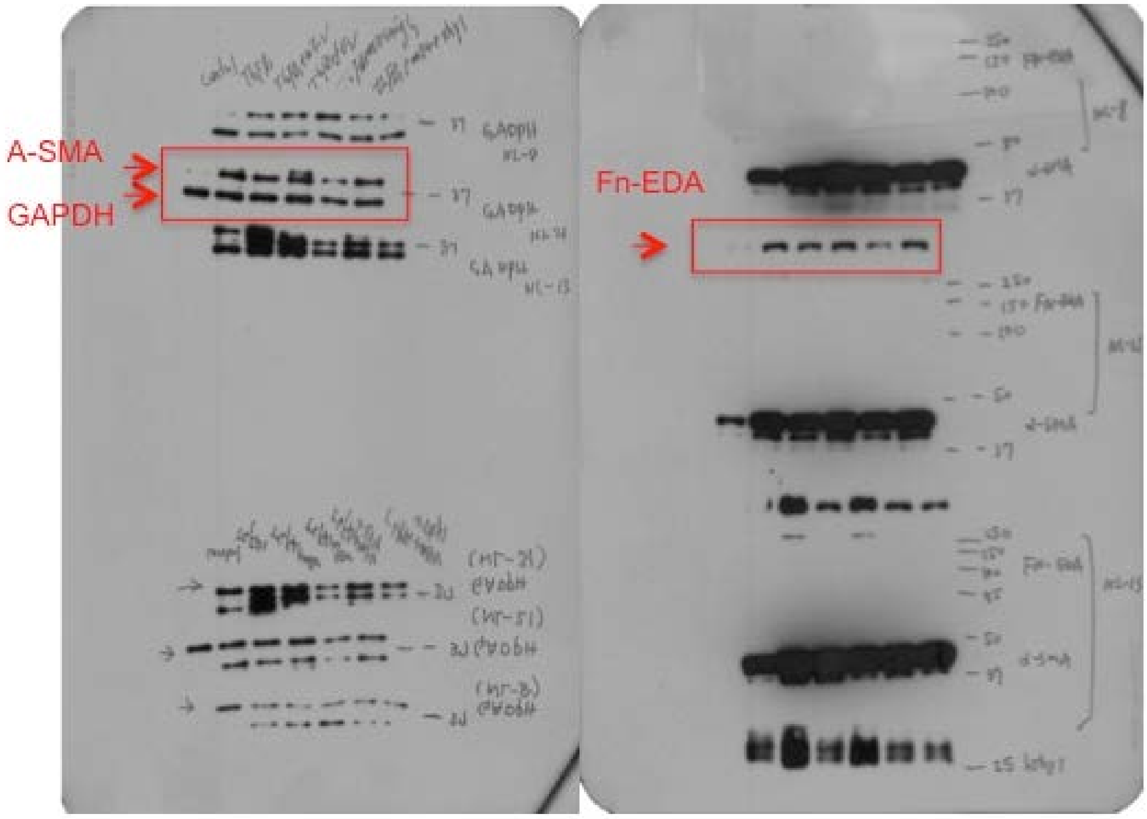

Supplemental Figure 10: Full western blot of Figure 1D

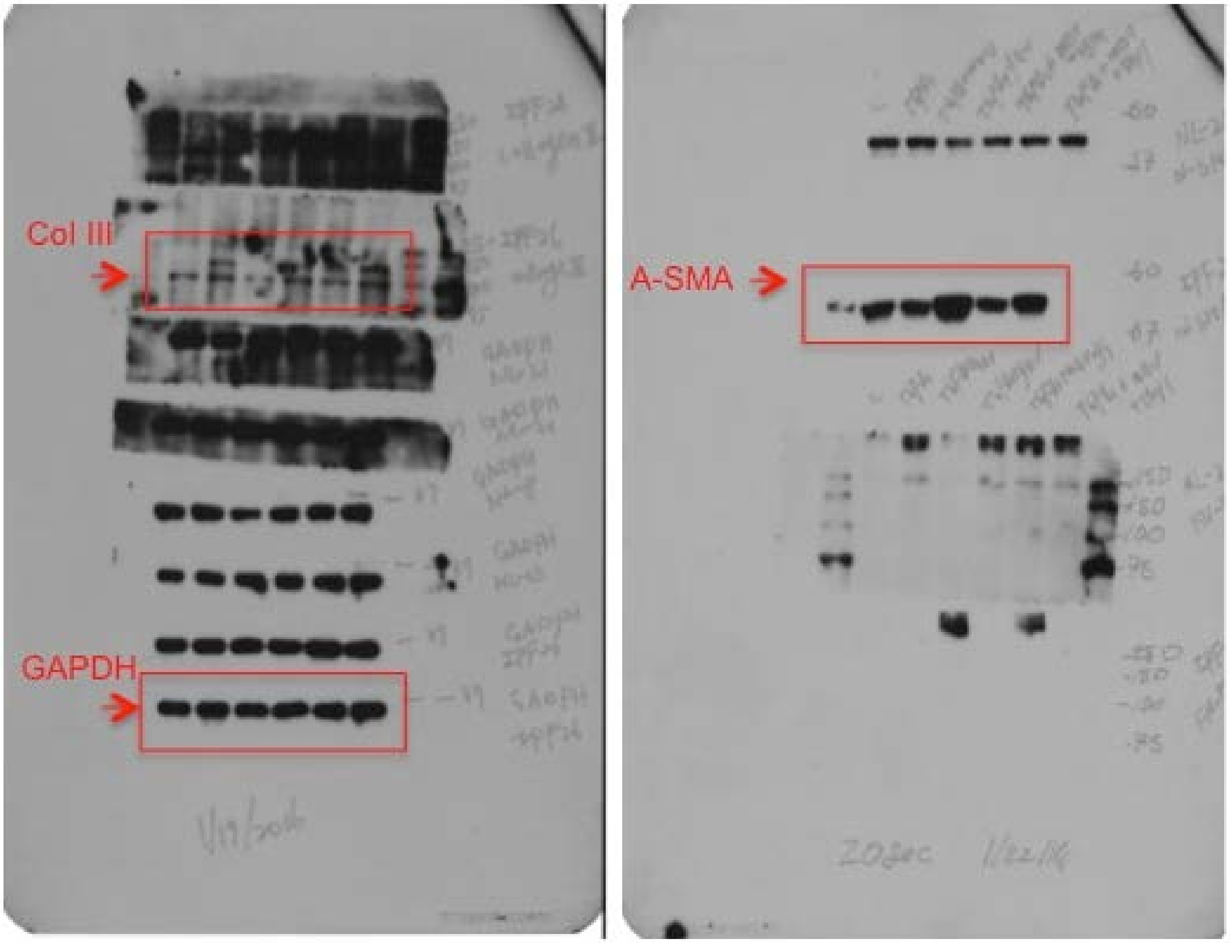

Supplemental Figure 11: Full western blot of Figure 3A

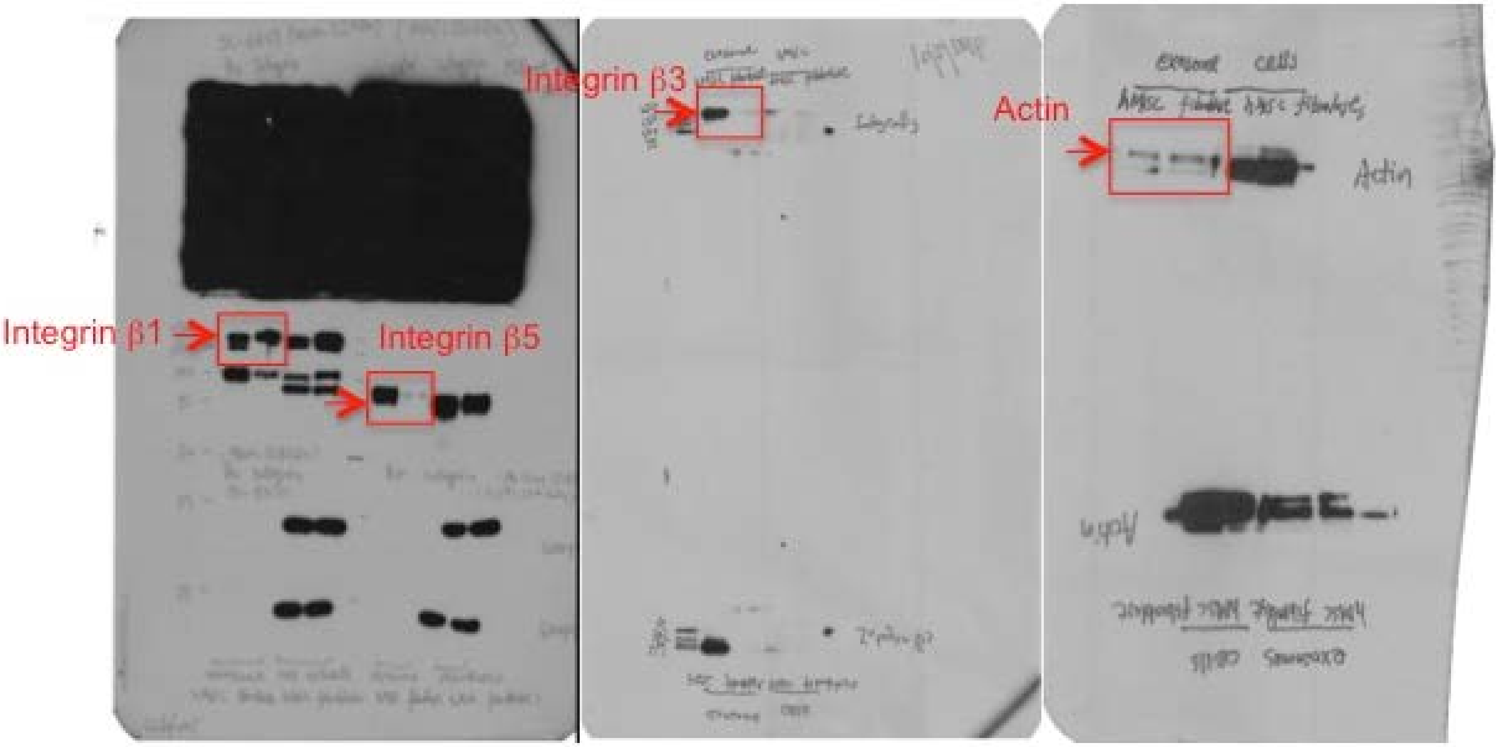

Supplemental Figure 12: Full western blot of Figure 5C

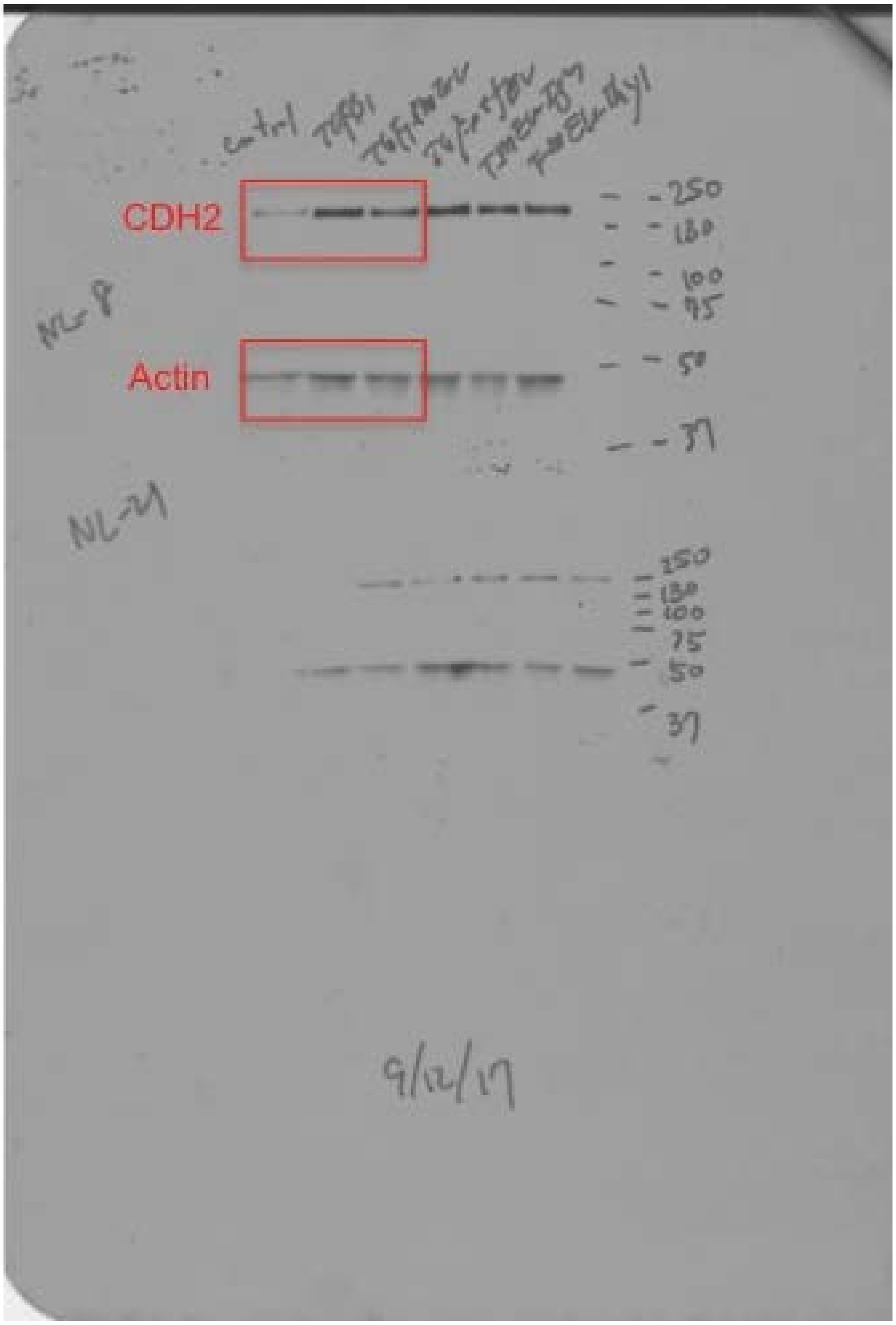

Supplemental Figure 13: Full western blot of Figure 5D

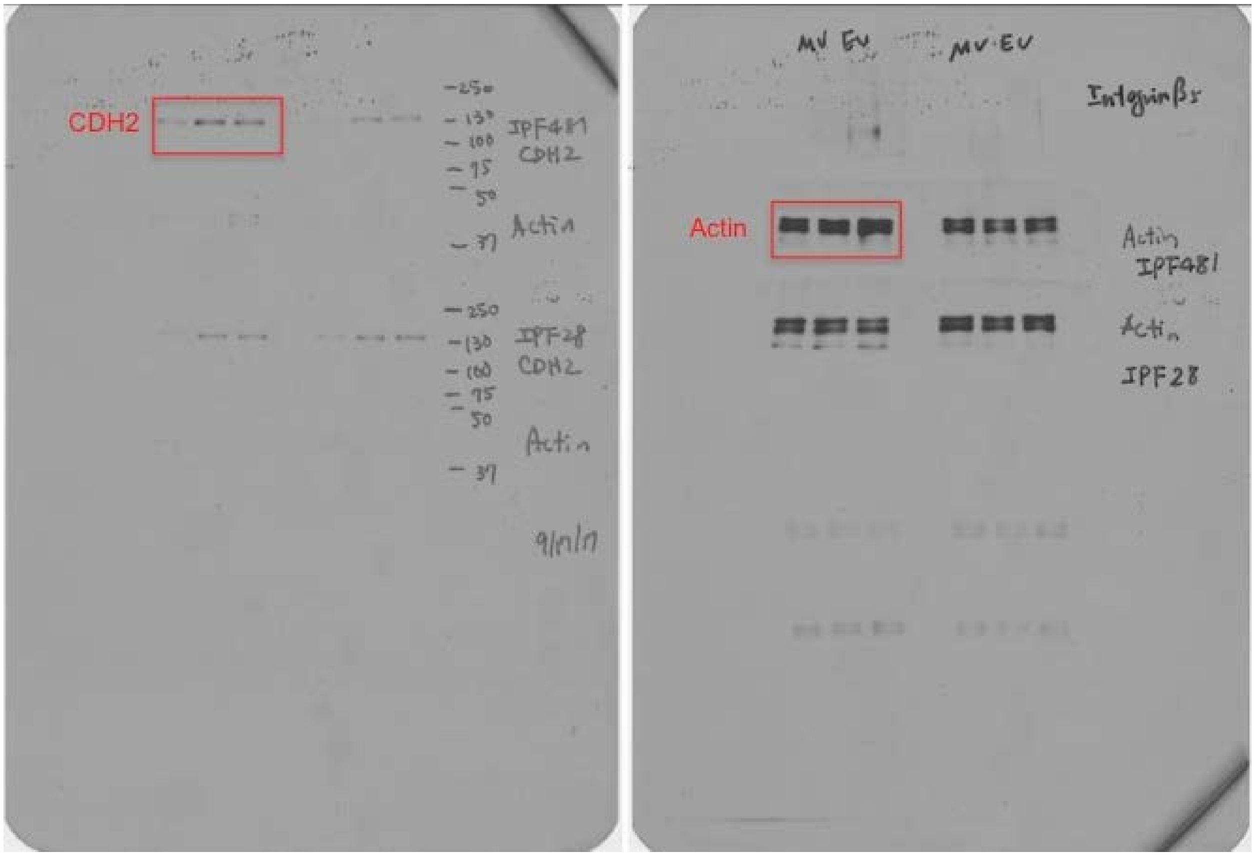

Supplemental Figure 14: Full western blot of Figure 5E

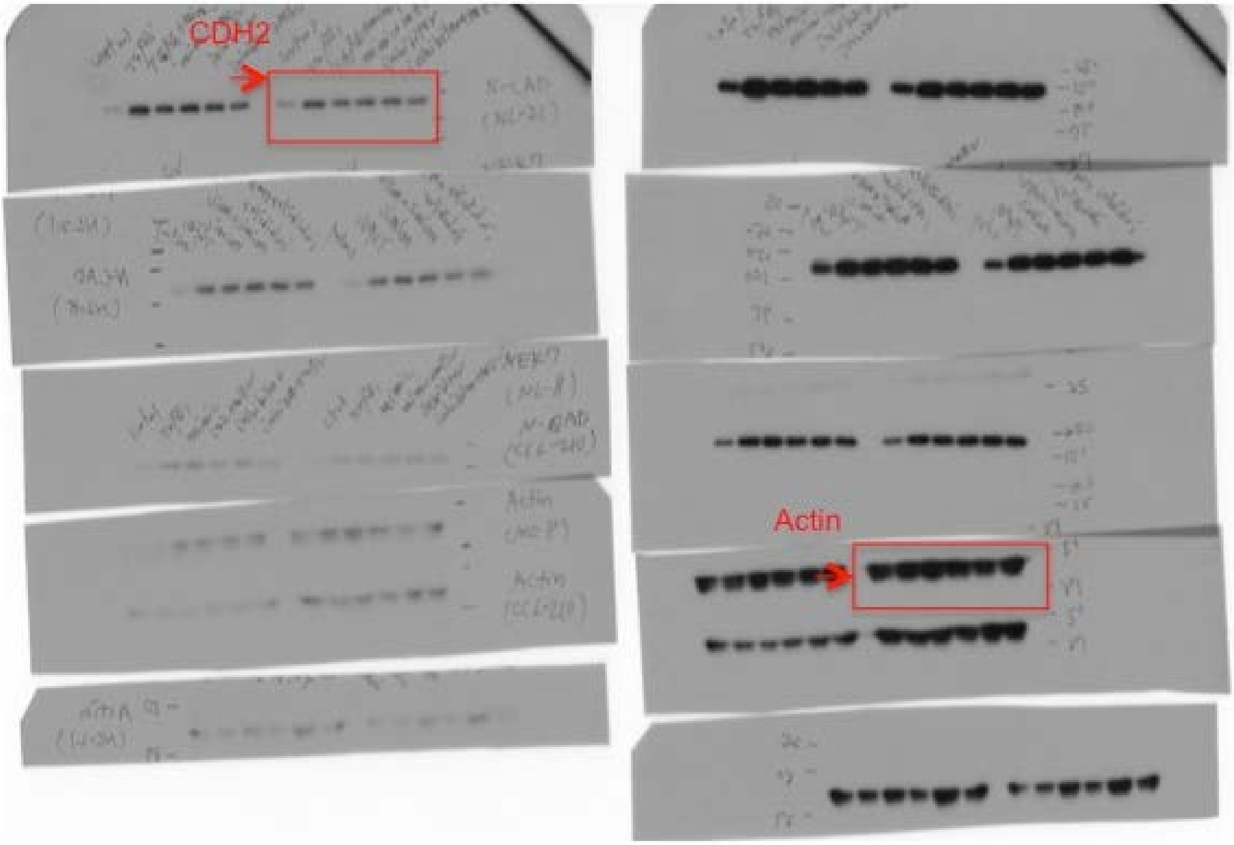

Supplemental Figure 15: Full western blot of Figure 5F

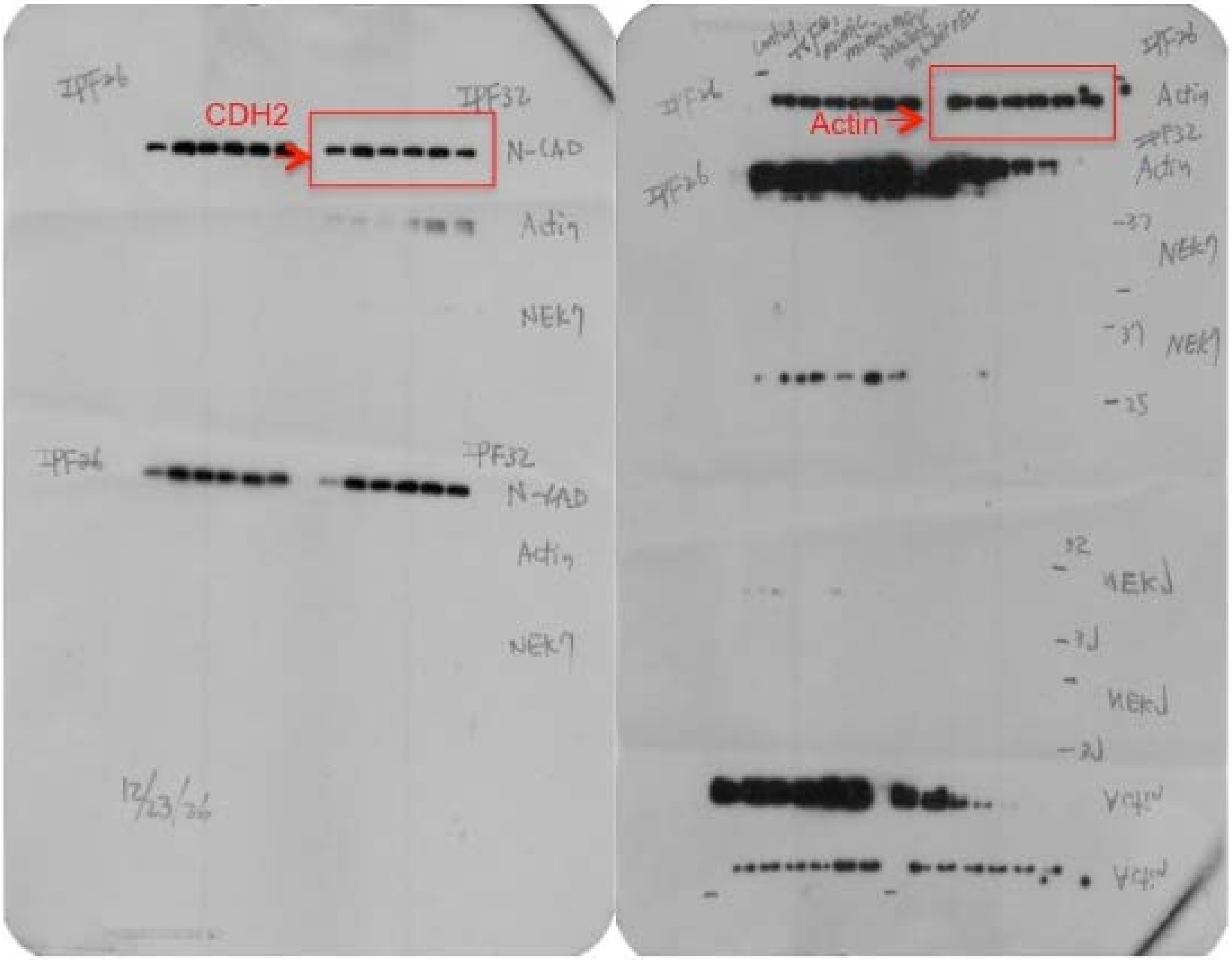

Supplemental Table 1: Human Gene primers for quantitative real-time PCR

Gene	Forward	Reverse
α-SMA	CCCATCTATGAGGGCTATGCCTTG	TCTCACGCTCAGCAGTAGTAACG
FN1- EDA	ATCGCCCTAAAGGACTGG	CATCCTCAGGGCTCGAGTAG
Col I A1	CAGCCGCTTCACCTACAGC	TTTTGTATTCAATCACTGTCTTGCC
Col IIIA1	ATTGCTGGGATCACTGGAGCAC	CCTGGTTTCCCACTTTCACCCTTG
N-cadherin	GAAGGACAGCCTCTTCTCAATGTG	TCACGGCATACACCATGCCATC
GADPH	TCAACGACCACTTTGTCAAGCTCA	GCTGGTGGTCCAGGGGTCTTACT

Supplemental Table 2: Upregulated genes in IPF fibroblasts compared to normal fibroblasts in GSE 40839 dataset

	GeneSymbol	LogFC	PVal	AdjPVal
206924_at	IL11	7.090434994	3.81E-11	5.31E-08
208937_s_at	ID1	5.707613809	3.10E-12	1.04E-08
217979_at	TSPAN13	4.881346137	5.62E-10	3.69E-07
207826_s_at	ID3	4.376495296	1.51E-10	1.37E-07
213725_x_at	XYLT1	4.246977785	1.38E-11	2.57E-08
204938_s_at	PLN	4.19084115	2.41E-07	2.99E-05
219773_at	NOX4	3.966922823	4.59E-12	1.16E-08
214240_at	GAL	3.940141434	4.74E-10	3.20E-07
222108_at	AMIGO2	3.87620306	9.04E-07	8.33E-05
221685_s_at	SPDL1	3.856927059	1.04E-08	2.96E-06
210602_s_at	CDH6	3.82589309	7.76E-07	7.42E-05
203951_at	CNN1	3.811646089	4.22E-06	0.000265356
202613_at	CTPS1	3.638495371	1.96E-12	8.72E-09
212670_at	ELN	3.587839693	2.76E-09	1.01E-06
210511_s_at	INHBA	3.453346562	1.20E-05	0.000581569
205132_at	ACTC1	3.444844055	1.56E-12	8.71E-09
209101_at	CTGF	3.315319847	6.63E-07	6.45E-05
206315_at	CRLF1	3.305765628	1.42E-10	1.37E-07
205700_at	HSD17B6	3.283115921	2.29E-10	1.82E-07
205680_at	MMP10	3.276200556	4.19E-07	4.48E-05
206116_s_at	TPM1	3.249829007	2.96E-09	1.06E-06
215813_s_at	PTGS1	3.245673886	3.15E-06	0.000216609
204589_at	NUAK1	3.226568574	1.29E-09	5.61E-07
200974_at	ACTA2	3.172489828	1.98E-05	0.000848992
201170_s_at	BHLHE40	3.067119514	7.24E-10	3.82E-07
202627_s_at	SERPINE1	3.061956074	2.61E-07	3.10E-05
204005_s_at	PAWR	3.061734675	1.54E-10	1.37E-07
219179_at	DACT1	3.052952965	3.38E-06	0.000228261
203243_s_at	PDLIM5	3.050642988	6.94E-06	0.000388376
213791_at	PENK	3.045164515	1.46E-09	5.90E-07
208447_s_at	PRPS1	2.969893944	3.36E-10	2.58E-07
204619_s_at	VCAN	2.927401334	2.42E-06	0.000175901
211981_at	COL4A1	2.922569447	1.75E-05	0.000774902
218051_s_at	NT5DC2	2.888741238	2.46E-07	3.01E-05
202620_s_at	PLOD2	2.883072241	2.42E-05	0.000988258
209031_at	CADM1	2.840798411	2.22E-05	0.000920474
212473_s_at	MICAL2	2.699791494	1.10E-06	9.54E-05

209016_s_at	KRT7	2.67783061	2.75E-07	3.21E-05
205428_s_at	CALB2	2.664635696	3.49E-09	1.18E-06
212099_at	RHOB	2.492368053	4.06E-07	4.37E-05
214807_at	PLXDC2	2.491978991	5.23E-07	5.36E-05
203418_at	CCNA2	2.465967084	4.19E-06	0.000264485
212572_at	STK38L	2.449124227	2.56E-07	3.07E-05
201309_x_at	NREP	2.411057263	9.80E-06	0.000508001
209209_s_at	FERMT2	2.387641259	6.44E-06	0.000365803
211756_at	PTHLH	2.345617788	3.91E-08	7.77E-06
205000_at	DDX3Y	2.311656239	8.62E-06	0.000462748
202779_s_at	UBE2S	2.302367943	6.99E-07	6.77E-05
218717_s_at	P3H2	2.294759685	3.16E-06	0.000216997
205925_s_at	RAB3B	2.294688221	2.56E-06	0.000183375
207463_x_at	PRSS3	2.249078411	1.91E-08	4.67E-06
221748_s_at	TNS1	2.234453079	1.59E-07	2.16E-05
207574_s_at	GADD45B	2.2309812	7.50E-08	1.25E-05
209542_x_at	IGF1	2.219001333	9.61E-08	1.50E-05
205266_at	LIF	2.141895025	5.74E-06	0.000332295
212256_at	GALNT10	2.120495162	2.23E-08	5.19E-06
203592_s_at	FSTL3	2.099353334	4.96E-08	9.21E-06
203085_s_at	TGFB1	2.098911877	6.22E-06	0.000356477
209765_at	ADAM19	2.065122896	7.70E-06	0.000422732
218350_s_at	GMNN	2.054670992	2.37E-06	0.000173512
205941_s_at	COL10A1	2.040829876	7.11E-10	3.82E-07
212489_at	COL5A1	2.037900571	2.24E-07	2.80E-05
218113_at	TMEM2	2.027455056	1.62E-07	2.19E-05
213252_at	SH3PXD2A	2.024974654	6.99E-08	1.18E-05
212985_at	APBB2	1.973610643	6.46E-07	6.34E-05
204352_at	TRAF5	1.958136333	2.24E-08	5.19E-06
209047_at	AQP1	1.956447548	4.91E-08	9.19E-06
204409_s_at	EIF1AY	1.902116379	9.00E-06	0.000476562
206007_at	PRG4	1.877714499	1.14E-06	9.87E-05
212345_s_at	CREB3L2	1.874431201	7.20E-06	0.000399248
205047_s_at	ASNS	1.868144816	2.77E-06	0.000195003
205117_at	FGF1	1.857282343	4.20E-07	4.48E-05
209681_at	SLC19A2	1.834064977	8.07E-09	2.50E-06
201044_x_at	DUSP1	1.816511243	3.67E-06	0.000243109
205100_at	GFPT2	1.812963913	2.17E-07	2.75E-05
203211_s_at	MTMR2	1.782384972	1.57E-05	0.00071584
210512_s_at	VEGFA	1.77634387	4.10E-06	0.000262485
200790_at	ODC1	1.769258157	8.87E-06	0.000472809

214606_at	TSPAN2	1.74749314	3.27E-07	3.76E-05
214927_at	ITGBL1	1.73932411	2.54E-07	3.06E-05
205324_s_at	FTSJ1	1.735291791	1.89E-07	2.46E-05
201136_at	PLP2	1.732555555	3.31E-07	3.77E-05
219142_at	RASL11B	1.730631932	2.24E-07	2.80E-05
218788_s_at	SMYD3	1.718552502	6.05E-09	1.90E-06
203440_at	CDH2	1.715109932	1.38E-07	1.96E-05
217591_at	SKIL	1.71122887	2.11E-06	0.000159592
201341_at	ENC1	1.687142444	4.00E-06	0.000258568
202241_at	TRIB1	1.678213281	2.72E-07	3.19E-05
219248_at	THUMPD2	1.667476553	5.09E-08	9.30E-06
210654_at	TNFRSF10D	1.667062505	1.68E-09	6.69E-07
210845_s_at	PLAUR	1.659436891	5.34E-08	9.52E-06
204749_at	NAP1L3	1.658544723	8.37E-06	0.000454942
220935_s_at	CDK5RAP2	1.649752348	2.82E-07	3.27E-05
200600_at	MSN	1.642878913	4.54E-06	0.000278747
209645_s_at	ALDH1B1	1.629930023	2.37E-06	0.000173512
212875_s_at	C2CD2	1.629529465	1.58E-07	2.16E-05
209822_s_at	VLDLR	1.603101814	9.94E-07	8.86E-05
203432_at	TMPO	1.595106868	1.23E-05	0.000594683
203743_s_at	TDG	1.593210912	1.60E-05	0.000722819
202363_at	SPOCK1	1.584239043	9.18E-08	1.46E-05
213793_s_at	HOMER1	1.577053527	1.69E-07	2.27E-05
206814_at	NGF	1.556680489	4.78E-06	0.000288622
204255_s_at	VDR	1.555856958	6.47E-06	0.00036659
221583_s_at	KCNMA1	1.554261538	6.30E-07	6.22E-05
219310_at	SYNDIG1	1.544555805	3.55E-08	7.25E-06
205807_s_at	TUFT1	1.533328399	5.79E-06	0.000333656
203232_s_at	ATXN1	1.530397006	1.47E-07	2.06E-05
214452_at	BCAT1	1.513881822	5.68E-08	9.97E-06
202133_at	WWTR1	1.479521696	5.37E-06	0.000315097
217356_s_at	PGK1	1.476063002	5.31E-06	0.000313193
221020_s_at	SLC25A32	1.474846939	2.07E-05	0.000877574
219366_at	AVEN	1.464102942	8.34E-06	0.000454536
212322_at	SGPL1	1.463748218	2.68E-06	0.000190179
209191_at	TUBB6	1.462491811	1.64E-06	0.000133076
204790_at	SMAD7	1.455395731	1.74E-06	0.000139273
218447_at	CMC2	1.451848306	2.30E-06	0.000170419
204241_at	ACOX3	1.451032921	8.59E-08	1.40E-05
201262_s_at	BGN	1.431530473	3.79E-06	0.000249214
217875_s_at	PMEPA1	1.424639901	4.22E-07	4.48E-05

213798_s_at	CAP1	1.418631664	9.80E-08	1.52E-05
58780_s_at	ARHGEF40	1.415990365	3.90E-06	0.000253551
200636_s_at	PTPRF	1.413533215	2.32E-06	0.000171256
203896_s_at	PLCB4	1.40987197	5.73E-06	0.000332295
209447_at	SYNE1	1.406861372	1.54E-07	2.13E-05
201947_s_at	CCT2	1.400127209	3.42E-08	7.05E-06
204462_s_at	SLC16A2	1.395611833	1.88E-07	2.46E-05
204136_at	COL7A1	1.388234943	1.32E-05	0.000627786
214121_x_at	PDLIM7	1.388159002	2.16E-07	2.75E-05
221539_at	EIF4EBP1	1.377303553	2.11E-05	0.000893329
205401_at	AGPS	1.372437785	2.80E-06	0.000196148
204023_at	RFC4	1.368179424	4.11E-06	0.000262557
207390_s_at	SMTN	1.366754027	2.56E-06	0.000183375
201577_at	NME1	1.35225739	1.24E-06	0.000105385
221658_s_at	IL21R	1.350470196	2.88E-06	0.000199254
218368_s_at	TNFRSF12A	1.343404738	4.17E-06	0.000264485
217755_at	HN1	1.338367277	1.16E-06	9.89E-05
218177_at	CHMP1B	1.336659852	5.53E-06	0.000321565
201841_s_at	HSPB1	1.336446886	1.53E-05	0.000701703
201516_at	SRM	1.322904373	1.12E-05	0.000551548
217841_s_at	PPME1	1.31764313	4.70E-08	8.96E-06
206176_at	BMP6	1.314689157	1.09E-07	1.64E-05
200712_s_at	MAPRE1	1.311760106	9.82E-09	2.88E-06
207265_s_at	KDELR3	1.311154549	1.95E-05	0.000838263
202401_s_at	SRF	1.306984347	1.52E-05	0.000697376
219806_s_at	SMCO4	1.305955767	1.27E-05	0.000608988
211023_at	PDHB	1.301160679	3.57E-07	3.97E-05
212070_at	ADGRG1	1.298727186	2.00E-06	0.000154027
208614_s_at	FLNB	1.282000369	9.92E-07	8.86E-05
201389_at	ITGA5	1.279771743	5.30E-06	0.000313193
208905_at	CYCS	1.265162395	8.42E-06	0.000455145
200830_at	PSMD2	1.262104059	1.72E-06	0.000138884
212923_s_at	PXDC1	1.239220385	4.07E-06	0.000261439
200808_s_at	ZYX	1.234426946	9.84E-06	0.000508001
214595_at	KCNG1	1.230642748	2.46E-08	5.66E-06
220173_at	BBOF1	1.209308093	8.73E-08	1.41E-05
202267_at	LAMC2	1.200649208	1.02E-05	0.000520331
213170_at	GPX7	1.186464595	8.77E-06	0.000469767
209526_s_at	HDGFRP3	1.180822803	8.91E-06	0.00047286
213571_s_at	EIF4E2	1.157166357	4.92E-06	0.00029467
215380_s_at	GGCT	1.156488866	3.68E-06	0.000243109

217503_at	STK17B	1.15317225	1.27E-07	1.83E-05
205071_x_at	XRCC4	1.149762075	3.40E-06	0.000228883
222020_s_at	NTM	1.142877742	1.98E-05	0.000849111
213011_s_at	TPI1	1.129913737	1.77E-05	0.000778897
218849_s_at	PPP1R13L	1.121651928	4.51E-06	0.00027818
208394_x_at	ESM1	1.119169305	1.73E-05	0.000769408
221840_at	PTPRE	1.113949041	9.12E-07	8.36E-05
220698_at	LOC79160	1.1124508	5.82E-07	5.84E-05
201980_s_at	RSU1	1.100904788	2.69E-06	0.000190179
207302_at	SGCG	1.097683995	1.37E-05	0.000643888
201096_s_at	ARF4	1.091960708	1.41E-05	0.000663335
221865_at	TMEM268	1.081594189	1.07E-07	1.61E-05
202777_at	SHOC2	1.080174272	2.28E-05	0.000943947
218223_s_at	PLEKHO1	1.076545563	3.52E-06	0.000236235
204334_at	KLF7	1.068740107	8.39E-07	7.92E-05
219677_at	SPSB1	1.061786037	6.38E-06	0.000363849
212157_at	SDC2	1.059098241	1.91E-06	0.000148092
36936_at	TSTA3	1.055147312	3.53E-06	0.000236532
212219_at	PSME4	1.042363554	1.47E-05	0.000679936
208737_at	ATP6V1G1	1.041786301	4.70E-07	4.94E-05
212722_s_at	JMJD6	1.041258476	2.12E-06	0.000159837
201844_s_at	RYBP	1.029563117	2.89E-07	3.33E-05
205194_at	PSPH	1.013339256	2.21E-05	0.000920472
218493_at	SNRNP25	1.007067793	5.39E-06	0.000315097
203238_s_at	NOTCH3	0.999521534	1.26E-05	0.000606066
205061_s_at	EXOSC9	0.997345021	4.57E-06	0.00027996
206538_at	MRAS	0.990390645	1.81E-05	0.000790823
204541_at	SEC14L2	0.988938466	9.69E-06	0.000505468
203484_at	SEC61G	0.986542372	1.19E-05	0.000577316
202418_at	YIF1A	0.985352057	9.69E-06	0.000505468
209949_at	NCF2	0.978740738	1.99E-07	2.55E-05
221619_s_at	MTCH1	0.972345348	9.49E-06	0.000497443
214462_at	SOCS6	0.968096339	1.86E-05	0.000807557
218394_at	ROGDI	0.956874606	2.65E-07	3.12E-05
202370_s_at	CBFB	0.946299882	8.54E-06	0.000459392
203252_at	CDK2AP2	0.944651216	1.62E-05	0.00073118
219480_at	SNAI1	0.929354291	2.19E-05	0.000914125
209314_s_at	HBS1L	0.92812387	1.32E-05	0.000627786
44783_s_at	HEY1	0.925402403	4.31E-06	0.000268422
201994_at	MORF4L2	0.918954334	1.98E-06	0.000152312
203821_at	HBEGF	0.91084591	1.08E-05	0.000539528

213549_at	PDZD8	0.869682042	3.29E-06	0.000223979
220765_s_at	LIMS2	0.867442353	4.29E-06	0.000268422
200709_at	FKBP1A	0.860282955	8.41E-06	0.000455145
203288_at	KIAA0355	0.852838593	1.01E-05	0.000516981
214719_at	SLC46A3	0.847614138	2.76E-06	0.000194595
221435_x_at	HYI	0.84571903	2.29E-05	0.000946787
204303_s_at	CTIF	0.82915835	3.42E-07	3.86E-05
205450_at	PHKA1	0.807156661	1.36E-05	0.000643245
220512_at	DLC1	0.771436321	1.30E-06	0.000107933
208813_at	GOT1	0.761998167	1.47E-05	0.000679936
208898_at	ATP6V1D	0.745594483	2.30E-06	0.000170419
212948_at	CAMTA2	0.731463257	1.62E-05	0.000729869
213278_at	MTMR9	0.7270413	1.17E-05	0.000572454
204471_at	GAP43	0.722534948	2.09E-05	0.000884486
221597_s_at	TMEM208	0.708646978	1.58E-05	0.00071715
204032_at	BCAR3	0.703378512	1.99E-05	0.000849111
216977_x_at	SNRPA1	0.700437781	1.34E-05	0.000635964
210156_s_at	PCMT1	0.687182703	7.23E-06	0.000399573
210872_x_at	GAS7	0.667745559	4.59E-06	0.00027996
206987_x_at	FGF18	0.663719527	1.81E-05	0.000790823
212129_at	NIPA2	0.657579501	7.00E-06	0.00038975
208968_s_at	CIAPIN1	0.621414768	6.79E-06	0.000381113
219474_at	C3orf52	0.597205085	1.82E-05	0.000792396
205442_at	MFAP3L	0.560029701	7.46E-06	0.000411709

Supplemental Table 3: Eight GO biological processes that are upregulated in IPF fibroblasts

Category	Description	Log P	Log (q-value)	Gene Symbols
GO0072359	Circulatory system development	-13.867	-9.622	ACTA2, ACTC1, AQP1, RHOB, CDH2, COL4A1, COL5A1, CTGF, FGF1, FKBP1A, HSPB1, ID1, ID3, IGF1, ITGA5, LIF, SMAD7, NOTCH3, SERPINE1, PLN, SGCG, SNAI1, SRF, TGFB1, TPM1, VEGFA, ADAM19, FGF18, SGPL1, ADGRG1, MICAL2, DLC1, PDLIM5, PPP1R13L, ESM1, JMJD6, HEY1, NOX4, TNFRSF12A
GO0007167	Enzyme linked receptor protein signaling pathway	-11.880	-8.111	ARF4, BMP6, COL4A1, CTGF, HBEGF, EIF4EBP1, FGF1, FKBP1A, HSPB1, ID1, IGF1, INHBA, ITGA5, LIF, SMAD7, NCF2, NGF, PLAUR, PTPRE, PTPRF, SDC2, SKIL, TGFB1, VEGFA, ZYX, SHOC2, FGF18, SGPL1, ATP6V1G1, FSTL3, FERMT2, ESM1, WWTR1, DACT1, ATP6V1D, PMEPA1, RASL11B
GO0061061	Muscle structure development	-8.560	-5.218	ACTC1, CDH2, HBEGF, FKBP1A, FLNB, ID3, IGF1, LIF, SMAD7, SGCG, SMTN, SRF, TGFB1, TPM1, VEGFA, HOMER1, MORF4L2, PDLIM5, MRAS, SYNE1, HEY1, NOX4, PLEKHO1, SMYD3
GO0072001	Renal system development	-7.619	-4.487	ACTA2, BMP6, COL4A1, FGF1, ID3, LIF, SMAD7, NOTCH3, ODC1, TGFB1, VEGFA, SGPL1, CRLF1, FSTL3, JMJD6, WWTR1
GO0001503	Ossification	-6.782	-3.815	BMP6, CBFB, COL10A1, VCAN, CTGF, ID1, ID3, IGF1, PENK, PTHLH, SNAI1, TGFB1, TUFT1, FGF18, PDLIM7, FSTL3, WWTR1
GO0043065	Positive regulation of apoptotic process	-6.712	-3.803	APBB2, RHOB, CTGF, DUSP1, ID3, INHBA, KCNMA1, GADD45B, NGF, PAWR, PLAUR, SKIL, TGFB1, VDR, STK17B, DLC1,

				MTCH1, NOX4, GAL, TNFRSF12A, CYCS
GO0034330	Cell junction organization	-6.707	-3.803	CDH2, CDH6, ITGA5, LAMC2, SMAD7, SNAI1, SRF, TGFB1, TNS1, VEGFA, DLC1, FERMT2, CADM1, LIMS2
R-HSA-1474244	Extracellular matrix organization	-6.484	-3.636	BGN, COL4A1, COL5A1, COL7A1, COL10A1, VCAN, ELN, ITGA5, LAMC2, MMP10, SERPINE1, PLOD2, SDC2, TGFB1, P3H2, APBB2, CTGF, FKBP1A, ID1, PRSS3, HSPB1, PLCB4
